# Supplementary material for: Deep learning and superoscillatory speckles empowered multimode fiber probe for in situ nano-displacement detection and micro-imaging
Source: Nat Commun. 2026 Jan 5;17:1174. doi: 10.1038/s41467-025-67942-8 (PMC12858975; doi:10.1038/s41467-025-67942-8)
Supplement: Supplementary file 1 — Supplementary Information [file 41467_2025_67942_MOESM1_ESM.pdf]

Supplementary information for

Deep learning and superoscillatory speckles empowered multimode fiber probe for in situ nano-displacement detection and micro-imaging

Lele Wang[1,2,†], Yiwei Zhang[3,†], Yibing Zhou[3], Yuan Meng[1], Zhengyang Lu[4], Pei Li[1,2], Hailong Zhang[1,2], Dan Li[1,2], Ping Yan[1,2], Qirong Xiao[1,2,*], Qiang Liu[1,2,*]

[1] Department of Precision Instrument, Tsinghua University, Beijing 100084, China

[2] State Key Laboratory of Precision Space-time Information Sensing Technology, Beijing 100084, China

[3] Weiyang College, Tsinghua University, Beijing 100084, China

[4] Weixian College, Tsinghua University, Beijing 100084, China

[†] These authors contributed equally: Lele Wang, Yiwei Zhang

* *Corresponding author: Qirong Xiao (xiaoqirong@mail.tsinghua.edu.cn)*

* *Corresponding author: Qiang Liu (qiangliu@tsinghua.edu.cn)*

Contents

Supplementary Text ... 3
Supplementary Note 1. Physical Model of multimode fiber at different displacements ... 3
Supplementary Note 2. Superoscillatory speckle patterns of multimode fiber ... 5
Supplementary Note 3. Architecture of Neural Network and Computing Configuration ... 8
Supplementary Note 4. Resolution and range of displacement detection ... 8
Supplementary Note 5. Continuous recognition capability of the system ... 10
Supplementary Note 6. Displacement detection and image reconstruction of optical fibers in different bending states ... 11
Supplementary Note 7. Displacement detection and material recognition of different metal materials ... 13
Supplementary Note 8. Compressed sampling of speckles for displacement detection and image reconstruction ... 15
Supplementary Note 9. Integrated System Architecture ... 17
9.1 Detection through the slit ... 17
9.2 Lens-free displacement detecting and imaging ... 17
9.3 Optimization of the air layer thickness ... 18
9.4 Optimization of the detection angle ... 19
9.5 Comparison of the proposed lensless system with the conventional multimode system ... 20
Supplementary Note 10. Reconstruction from Frequency Spectrum of the Diffuse Speckle Field ... 20
Supplementary Note 11. Optimizing imaging quality with the Displacement-Inverse Transmission Matrix Library（DITML） ... 21
Supplementary Note 12. 3D micro cross-section reconstruction by diffuse speckles ... 21
Supplementary Figures ... 23
Supplementary Table ... 52
References ... 53

Supplementary Text

Supplementary Note 1. Physical Model of multimode fiber at different displacements

To explore how the displacement of the target influences the transmission modes, a physical model is built to simulate the optical field propagation and coupling processes in multimode fiber (MMF).

The angular spectrum method is employed to calculate the propagation of the optical field output from the DMD to the fiber-end-ball[1]. The specific process involves performing a Fourier transform on the optical field to obtain its spectrum:

$$U_{1F} = \mathcal{F}(U_1) \tag{S1}$$

The spectrum of the light field U_2 in front of the fiber-end-ball is obtained by multiplying U_{1F} with the transfer function:

$$U_{2F} = U_{1F} \times H_{AS-Air} \tag{S2}$$

$$H_{AS-Air} = \exp\left(ikz\sqrt{1-(\lambda f_x)^2-\left(\lambda f_y\right)^2}\right) \tag{S3}$$

Where H_{AS-Air} represents the angular spectrum transfer function describing the light field propagation from the target pattern, through free-space transmission, to the front surface of the fiber ball lens, k is the wave number, z is the distance between the DMD and the fiber-end ball, λ is the wavelength, and f_x and f_y are the spatial frequency components in the x and y directions.

And then performing an inverse Fourier transform to obtain the optical field U_2 as:

$$U_2 = \mathcal{F}^{-1}(U_{2F}) \tag{S4}$$

The fiber-end ball is treated as a spherical lens with a fixed radius to determine the optical field entering the fiber as:

$$U_3 = U_2 \cdot \exp\left(ik\left(R+(n-1)\sqrt{R^2-(x^2+y^2)}\right)\right) \tag{S5}$$

Here, k is the wavenumber in vacuum, R is the radius of curvature of the fiber-end-ball, n is the refractive index of the ball material, and x and y are the coordinates in the plane.

The fiber-end-ball is treated as a thin lens. Similar to the previously described light propagation process, we obtain the optical field U_4 at a diffraction distance $d = 1.25mm$ after the ball. This diffraction distance is obtained using a microscope after the fiber-end-ball is fabricated

$$U_{3F} = \mathcal{F}(U_3) \tag{S6}$$

$$U_{4F} = U_{3F} \times H_{AS-Ball} \tag{S7}$$

$$H_{AS-Ball} = \exp\left(ikd\sqrt{1-(\lambda f_x)^2-\left(\lambda f_y\right)^2}\right) \tag{S8}$$

Where $H_{AS-Ball}$ characterizes the angular spectrum transfer function describing the light field propagation from the front surface of the fiber ball lens, through transmission inside the ball lens, to the rear surface (the input facet of the multimode fiber).

$$U_4 = \mathcal{F}^{-1}(U_{4F}) \tag{S9}$$

And the output field is then projected onto the LP mode coordinate basis of the fiber to obtain the projection coefficients.

$$C_{i(m,n)} = \frac{\int_0^\infty \int_0^\infty \mathrm{LP}_{mn}^*(x,y)U_4(x,y)dxdy}{\int_0^\infty \int_0^\infty \mathrm{LP}_{mn}^*(x,y)\mathrm{LP}_{mn}(x,y)dxdy} \tag{S10}$$

Where $\mathrm{LP}_{mn}(x,y)$ represents the distribution of linearly polarized mode fields with angular mode order and radial mode order of m and n, respectively. i represents the total mode order, which is obtained by arranging these LP mode fields in the order of the cutoff frequency. In this experiment, the first 1000 modes are taken into consideration. Project the input mode field to the first 1000 LP mode fields to get the mode coefficients $C_1 \sim C_{1000}$. In order to more intuitively evaluate the energy share of the input light field decomposed into each mode, further calculations were performed to obtain the energy percentage factor:

$$P_I(i) = \frac{|C_i|^2 \int_0^\infty \int_0^\infty \mathrm{LP}_{mn}^*(x,y)\mathrm{LP}_{mn}(x,y)dxdy}{I_{U_4}} \tag{S11}$$

Where, I_{U_4} is the total input energy of U_4,

$$I_{U_4} = \int_0^\infty \int_0^\infty U_4^*(x,y)U_4(x,y)dxdy \tag{S12}$$

Fig. S1 shows simulation architecture for optical field evolution of multimode fiber probes. Simulation results of the physical model at different displacements (z=0mm, z=10mm, z=20mm, z=30mm) are shown in Fig. S2.

In order to characterize the overall mode distribution and the higher-order mode share of different displacement, the normalized average mode order is defined:

$$O_n = \frac{\sum_i P_I(i) \times i}{\sum_i P_I(i)} \tag{S13}$$

To further investigate the patterns under large displacement ranges, we used wafer microscopic images to simulate within a large displacement measurement range of 0–10 mm. Sampling points were taken at 1 mm intervals, and scatter plots of the normalized mode order, the percentage sum

of first 100 LP modes, and percentage sum of first 1000 LP modes were plotted along with fitted curves, as shown in Fig. S3. It can be observed that, although fluctuations exist in the normalized mode order and the percentage sum of first 100 and first 1000 LP modes at certain positions, an overall monotonic trend is evident. Within the large displacement range, the proportions of both the first 100 and the first 1000 LP modes exhibit an increasing trend, while the normalized average mode order shows a decreasing trend. These results are consistent with Fig. 2f. The normalized modes coefficient are also shown in Table S1.

Overall, we wish to emphasize that through modal decomposition of the optical field under different displacements, a certain inherent relationship exists between the displacement of the detection target and the mode composition within the multimode fiber, ultimately influencing the distribution of the output speckle field. Although some analyzed variables may exhibit fluctuations or even opposite trends under small displacements, this intrinsic relationship remains present. This part of the study provides a physical basis and a degree of interpretability for the displacement recognition neural network discussed later in the manuscript.

Supplementary Note 2. Superoscillatory speckle patterns of multimode fiber

The optical superoscillatory phenomenon creates subwavelength structures in the phase distribution of light by constructing multiple interferences of the light field. This phenomenon enables the resolution of light to surpass the diffraction limit, achieving nanoscale or even atomic-scale. It is currently used as "optical ruler" or for designing super-oscillation lenses[2] to achieve super-diffraction imaging or displacement measurements.

Superoscillation also exists in speckle fields[3]. In experiments, we observed the superoscillatory phenomenon in the speckle field of multi-mode fiber, which demonstrates the capability of the proposed system in nano-displacement measurement. Since there is no interference light path in our system, we solved the phase of the speckle light field using the Transport of Intensity Equation(TIE)[4].

Specifically, we assume that the speckle light field output from the fiber is a monochromatic light beam freely transmitted along the z-axis, and its complex amplitude can be represented as follows:

$$U(x,y,z) = \sqrt{I(x,y,z)}\exp[\,i\varphi(x,y,z)] \tag{S14}$$

Where $U(x,y,z)$ is the complex amplitude of the light field, $I(x,y,z)$ is the intensity of the light field, and $\varphi(x,y,z)$ is the phase of the light field. Under the paraxial approximation, the propagation of the light field satisfies the following parabolic equation:

$$(2ik\partial_z + \Delta)U(x,y,z) = 0 \tag{S15}$$

Where $\partial_z = \partial/\partial z$ represents the change in intensity distribution along the z-axis, $\nabla = (\partial/\partial x, \partial/\partial y)$ denotes the gradient operator, and $\Delta = \nabla^2 = \partial^2/\partial x^2 + \partial^2/\partial y^2$ represents the two-dimensional Laplacian operator. k is the wave number, which satisfies k=2π/λ where λ is the

wavelength. The one-dimensional partial derivative of $\mathrm{U}(x,y,z)$ is taken with respect to the direction of (x,y,z).

$$\begin{aligned}\partial_x U(x,y,z) &= \frac{1}{2} I^{-1/2}\cdot \partial_x I\cdot e^{i\varphi} + I^{1/2}\cdot e^{i\varphi}\cdot i\cdot \partial_x\varphi \\ \partial_y U(x,y,z) &= \frac{1}{2} I^{-1/2}\cdot \partial_y I\cdot e^{i\varphi} + I^{1/2}\cdot e^{i\varphi}\cdot i\cdot \partial_y\varphi \\ \partial_z U(x,y,z) &= \frac{1}{2} I^{-1/2}\cdot \partial_z I\cdot e^{i\varphi} + I^{1/2}\cdot e^{i\varphi}\cdot i\cdot \partial_z\varphi\end{aligned} \tag{S16}$$

The two-dimensional partial derivatives of $U(x,y,z)$ are taken with respect to the x and y directions, respectively,

$$\begin{aligned}\partial_x^2 U(x,y,z) &= -\frac{1}{4} I^{-\frac{3}{2}}\cdot \partial_x I\cdot e^{i\varphi} + \frac{1}{2} I^{-\frac{1}{2}}\cdot \partial_x^2 I\cdot e^{i\varphi} + I^{-\frac{1}{2}}\cdot e^{i\varphi}\cdot i\cdot \partial_x I\cdot \partial_x\varphi \\ &\quad - I^{\frac{1}{2}}\cdot e^{i\varphi}\cdot (\partial_x\varphi)^2 + I^{\frac{1}{2}}\cdot e^{i\varphi}\cdot i\cdot \partial_x^2\varphi \\ \partial_y^2 U(x,y,z) &= -\frac{1}{4} I^{-\frac{3}{2}}\cdot \partial_y I\cdot e^{i\varphi} + \frac{1}{2} I^{-\frac{1}{2}}\cdot \partial_y^2 I\cdot e^{i\varphi} + I^{-\frac{1}{2}}\cdot e^{i\varphi}\cdot i\cdot \partial_y I\cdot \partial_y\varphi \\ &\quad - I^{\frac{1}{2}}\cdot e^{i\varphi}\cdot \left(\partial_y\varphi\right)^2 + I^{\frac{1}{2}}\cdot e^{i\varphi}\cdot i\cdot \partial_y^2\varphi\end{aligned} \tag{S17}$$

Calculate the conjugate function $U^*(x,y,z)$ of $U(x,y,z)$,

$$U^*(x,y,z) = \sqrt{I(x,y,z)}\exp[-i\varphi(x,y,z)] \tag{S18}$$

Multiplicate the $U^*(x,y,z)$ with left of formula (S15), we obtain:

$$\begin{aligned}&ik\cdot \partial_z I - 2Ik\cdot \partial_z\varphi - \frac{1}{4} I^{-1}\cdot \partial_x I + \frac{1}{2}\partial_x^2 I + i\cdot \partial_x I\cdot \partial_x\varphi - I(\partial_x\varphi)^2 + \\ &I\cdot i\cdot \partial_x^2\varphi - \frac{1}{4} I^{-1}\cdot \partial_y I + \frac{1}{2}\partial_y^2 I + i\cdot \partial_y I\cdot \partial_y\varphi - I\left(\partial_y\varphi\right)^2 + I\cdot i\cdot \partial_y^2\varphi = 0\end{aligned} \tag{S19}$$

Multiplicate the $U(x,y,z)$ with left of formula (S17), we obtain:

$$\begin{aligned}&-ik\cdot \partial_z I - 2Ik\cdot \partial_z\varphi - \frac{1}{4} I^{-1}\cdot \partial_x I + \frac{1}{2}\partial_x^2 I - i\cdot \partial_x I\cdot \partial_x\varphi - I(\partial_x\varphi)^2 - \\ &I\cdot i\cdot \partial_x^2\varphi - \frac{1}{4} I^{-1}\cdot \partial_y I + \frac{1}{2}\partial_y^2 I - i\cdot \partial_y I\cdot \partial_y\varphi - I\left(\partial_y\varphi\right)^2 - I\cdot i\cdot \partial_y^2\varphi = 0\end{aligned} \tag{S20}$$

Subtract equation (S19) from equation (S20) and simplify, we obtain:

$$k\cdot \partial_z I + \left(\partial_x I\cdot \partial_x\varphi + \partial_y I\cdot \partial_y\varphi\right) + I\left(\partial_x^2\varphi + \partial_y^2\varphi\right) = 0 \tag{S21}$$

Further simplify the above expression, we obtain:

$$-k \cdot \partial_z I = \nabla I \cdot \nabla\varphi + I\Delta\varphi = \nabla \cdot (I\nabla\varphi) \tag{S22}$$

The above equation is the derived TIE, which indicates that after clarifying the intensity distribution and the intensity differential along the optical axis, the next step is to solve the second-order partial differential equation. Ultimately, this will provide the specific phase distribution information on the plane at position z_0. Generally, by capturing two or more defocused images, the axial derivative can be predicted using the finite difference method.

Several methods are commonly used to solve the intensity transfer equation, including Fourier methods, multigrid methods, Green's function methods, and methods that extend it into polynomials. Here, we adopt the Fourier method for solving this equation. This method can be applied in two cases: one where the light field intensity distribution is uniform, and another where it is non-uniform. Clearly, for the speckle field of MMF, the intensity distribution is non-uniform. Paganin et al. extended the Fourier method by introducing auxiliary functions into the equation solving process. The phase obtained from this solution is as follows:

$$\varphi(x, y) = -\mathcal{F}^{-1} q_\perp^2 \mathcal{F}\{\nabla_\perp \cdot [I^{-1}(x, y)\psi(x, y)]\} \tag{S23}$$

Where,

$$\psi(x, y) = \mathcal{F}^{-1} q_\perp^2 \mathcal{F}\left[\frac{k\partial I(x, y)}{\partial z}\right], q_\perp^2 = f_x^2 + f_y^2 \tag{S24}$$

f_x and f_y represent the coordinates in the frequency domain.

In equation (S23), there is the term for the axial derivative of intensity. Typically, the axial derivative of intensity is approximated using the finite difference method.

$$\left.\frac{\partial I(x, y, z)}{\partial z}\right|_{z=z_0} \approx \frac{I(x, y, z_0 + \Delta z) - I(x, y, z_0 - \Delta z)}{2\Delta z} \tag{S25}$$

Where z_0 is the propagation distance of the speckle field, $I(x, y, z_0 + \Delta z)$ and $I(x, y, z_0 - \Delta z)$ are the intensity distributions at the recording planes $z_0 + \Delta z$ and $z_0 - \Delta z$, respectively, and Δz is the distance between the two recording planes.

In the experiment, we mounted the camera at the fiber output end onto a nanometer displacement stage. At a distance of 4.8 mm from the fiber end face, with a 10 nm movement interval, we recorded two speckle light fields. After cropping and processing, the two speckle patterns are shown in Fig. S4a and Fig. S4b.

Using these two speckle intensity distribution patterns at different propagation distances and the TIE, we calculated the phase distribution of the speckle field near 4.8 mm, as shown in Fig. S4d. Taking the gradient of the phase, $k = \Delta\varphi$, we obtain the magnitude (Fig. S4e) and direction distribution (Fig. S4f) of the phase field gradient.

It can be observed that the phase field exhibits rapid gradient changes. Based on the magnification of the optical path and the diameter of the optical fiber output face, it can be calculated that the size of the phase rapid variation patterns is approximately 300 nm. What we want to note is that further improvements in camera resolution and optical path magnification can allow the observation of even smaller rapid variation patterns[5]. The superoscillation phenomenon in multi-mode fiber speckle demonstrates the capability of multi-mode fibers for nano-displacement measurements and holds potential for super-diffraction-limit imaging and metrology.

Supplementary Note 3. Architecture of Neural Network and Computing Configuration

The diffuse speckle field, which is compressed into 128×128 pixels, is fed into the VGG displacement recognition network. First, it passes through a 3×3 convolutional layer, followed by a hyperbolic tangent activation layer and a 2×2 maximum pooling layer. This setup is iterated, doubling the channel depth from 32 successively to 256. The same kernel size, activation, and pooling are maintained at each step. The output is then flattened and fed into a fully connected (FC) layer of size 1×16384. The tanh activation function is applied. The two subsequent FC layers progressively reduce the number of elements from 16384 to the number of displacement samples (N). The specific network architecture is shown in Fig. S5a. The model is trained with 50 epochs, Adam is chosen as the optimizer, and the learning rate is set to 1×10^{-3}.

The inverse transmission matrix (ITM) optimization network is trained for image reconstruction. Since the transmission matrix is a complex matrix and the speckle field acquired by the camera is a real intensity field. The square root of the acquired speckles I (intensity data) is extracted as the input X (amplitude data) to the model. The imaginary part of the matrix needs to be filled with 0. Then, we initialize the inverse transfer matrix by the "Glorot Uniform" method[6]. Then multiply it with the speckle matrix. The modulo square of the resulting matrix from the multiplication is the vector of the reconstructed image. By solving the cosine similarity of the reconstructed image and the real image, the inverse transmission matrix is further optimized. The specific network architecture is shown in Fig. S5b. The model is trained with 105 epochs, Adam is chosen as the optimizer, the batch size is set to 36, and the learning rate is set to 1×10^{-5}.

The dataset used in the experiments are cropped square natural scene grayscale images[7]. In the displacement detection experiments, we collect 4000 sets of patterns at each displacement, dividing 70% of them into a training set, 10% into a validation set, and 20% into a test set. In the imaging experiments, at each displacement we collect 8000 sets of image-speckle pairs, dividing 7200 of them into a training set, 400 into a validation set, and 400 into a test set. The results shown in the manuscript and supplementary material are selected from the test set output. We train the data on the Colab platform[8] provided by Google. The graphics cards used are T4 and A100.

Supplementary Note 4. Resolution and range of displacement detection

To further validate the capability of nano-displacement detection of our system, we designed a series of experiments. We use a nano-electric displacement stage (COREMORROW, P18.X300S & New focus, Model 8751-C) employing an intelligent pico-motor driver for closed-loop pico-motor to control small displacements of the DMD. Fig. S6 shows the experimental setup at the probe end. First, we test the accuracy of displacement detection at different intervals with New

focus's nano-electric displacement stage, specifically at 508nm, 0.01mm, 0.05mm, and 0.2mm. To eliminate potential confounding variables besides distance, the experimental environment and the parameters of data analysis are strictly controlled. We standardized the series of experiments with a uniform set of ground truth images. The average intensities of speckle fields at different positions remain consistent.

The output speckle corresponding to the input image is captured and the input image is repeated 5 times for 5 displacements at each resolution (for instance, displacement of 0mm, 0.01mm, 0.02mm, 0.03mm, and 0.04mm for the 0.01mm resolution). Laser power also needs to be adjusted, in order that the maximum grey value at the center of the speckle is consistent at different positions. To avoid introducing biases during image processing, all speckle images were processed at fixed radius and size before they are input into VGG network, all parameters of which remain consistent. With a displacement interval of 508 nm, our network is still able to achieve 100% detection accuracy.

Further, we replace the displacement stage carrying the probe target with COREMORROW, P18.X300S to verify the nano-displacement detection capability of the proposed system. Using this high-precision displacement stage, we tested the displacement detection capability of the system in closed-loop mode at 100 nm and 10 nm resolutions. The entire test system is placed on an optical vibration isolation platform. The whole system realizes automated acquisition to ensure that there is no human interference. Fig. S7 demonstrates the displacement detection results at different resolutions. It can be seen that even at a displacement of 10 nm, the average accuracy of displacement recognition can still reach 99.27%. To highlight smaller values, we changed the color mapping relationship.

In order to verify the practical applicability of the system and to overcome the round-trip vibration of the target, we conducted validation experiments at a displacement resolution of 100 nm. Specifically, we moved the displacement stage from 0-400 nm at a displacement interval of 100 nm and collected 4000 sets of speckles at each displacement position. After that, we reversed the displacement stage and retreated from 400 nm to 0 nm. Similarly, 4000 sets of speckles were collected at 100 nm intervals. Since the return accuracy of the displacement stage cannot reach 10 nm, we performed the return experiments at 100 nm resolution. Fig. 3e in the manuscript shows the results of the backward experiment at 100 nm resolution, where the displacement recognition accuracy can reach 99.39%.

Regarding the range of the system, we first tested the system using a mechanical displacement stage. Specifically, we mounted the DMD on the mechanical displacement stage and collected data at different displacements at 0.5 mm intervals. Similarly, 4000 sets of speckles were collected at each position. 100% displacement recognition accuracy is achieved. It is proved that the proposed system can detect in the range of 2mm. Then we collected 16 and 20 groups of data at 508nm and 10nm, respectively. The processing capability of the network for more sampling points has been verified. Fig. S8b and Fig. S8c show the confusion matrices for these two experiments. The displacement recognition accuracy can reach 99.88% and 98.42% for the two experimental configurations, respectively. Additionally, although neural network-based methods generally require large amounts of data and training costs, our approach achieves an accuracy of 95.48% using just 100 speckles per group as the training set. Fig. S8d and S8e presents the probability

statistics of each sampled group under the correct displacement label with 500 datasets and 100 datasets. Furthermore, we conducted high-resolution (10 nm) testing across the broad displacement range of 0–990 nm. The confusion matrix, shown in Fig. S9, still achieves an accuracy of 97.8%, demonstrating the robustness of our approach to larger-range displacement classification.
For higher sampling densities and ranges, higher configurations of computing devices may be required to meet the memory requirements. Alternatively, high accuracy and large range displacement detection can be achieved by compressing the samples to reduce the memory requirements.

In addition to the two strategies mentioned above (Training with Small Datasets and Speckle Size Compression) we can also achieve high-resolution measurements across a wide range using the Multi-interval Partitioned Recognition method. This approach does not require large, complex networks, nor does it necessitate increasing the number of neurons or computational costs.

The hierarchical classification strategy to effectively balance the resolution and range. Specifically, we first perform coarse classification by grouping adjacent displacements (e.g., 10 neighboring classes) into a super-class to locate the rough displacement interval, followed by fine classification within the identified interval to achieve high-resolution discrimination. The overall process concept is shown in Fig. S10a. This two-stage approach significantly reduces the output dimensionality and computational demand while maintaining high accuracy.

As a demonstration, we conducted additional experiments and collected experimental data over a large range of 0–500 nm at a high resolution of 10 nm, totaling 50 displacement sampling points. Following the above strategy, we first performed coarse classification by grouping every five adjacent displacement points into one category. After completing this initial step, the corresponding interval was identified, and a second displacement recognition network was trained using fine-classification data to conduct detailed classification. Fig. S10b presents the confusion matrices for the coarse classification (each super-category containing five classes) and two corresponding fine-classification tasks. As shown in Fig. S10c, these results collectively validate the effectiveness of the proposed hierarchical strategy.

In scenarios requiring even higher resolution and larger ranges, the number of hierarchical levels can be further increased to implement multi-stage recognition. This approach compresses the number of neurons in the network and reduces computational cost at the expense of certain time overhead, thereby achieving high-accuracy displacement recognition over extensive ranges and at high resolutions.

Supplementary Note 5. Continuous recognition capability of the system

Although the main text only demonstrated discrete displacement recognition results obtained using classified-like networks, neural networks based on superoscillatory speckle can output continuous displacement values.To achieve higher resolution and continuous displacement measurement, we reformulated the original discrete classification problem into a regression task, allowing the network to output continuous displacement values rather than being restricted to fixed class labels.

To achieve this, we propose two strategies here: Gaussian Label Regression and encoded rectangular patterns.

Firstly, we adopted a method known as Gaussian Label Regression, where the conventional one-hot labels are replaced with soft Gaussian-distributed labels centered on the true displacement. This introduces inter-class continuity and allows the model to learn a smoother and more generalizable output space.

During training, we employed a hybrid loss function. The KL divergence measures the difference between the predicted probability distribution and the Gaussian-shaped target, while the mean squared error (MSE) penalizes the difference between the predicted expected displacement and the ground-truth value. As shown in Fig. S11 this strategy enables the network to produce continuous outputs.

To evaluate the method's generalization capability, we divided the displacement classes by parity: even-indexed classes (0, 20, 40 nm…) were used for training, a small portion of odd-indexed samples (10, 30 nm…) were included for validation, and the remaining odd classes were used as the test set. As shown in Fig. S12, the model accurately predicted displacements even for the test classes not seen during training confirming the method's effectiveness. The prediction accuracy for even classes is relatively high, while that for odd classes is lower. There remains room for improving the choice of Gaussian hyperparameters and loss functions in this method.

The second method encodes speckle patterns corresponding to different displacements with rectangular patterns at varying positions and trains a U-Net network, as illustrated in Fig. S13. Specifically, speckle patterns at each displacement are mapped to rectangular patterns at different locations, where the rectangle's position follows an S-shaped trajectory as displacement increases. After training, the network takes a speckle pattern as input and outputs the corresponding pattern. By calculating the centroid of the output pattern and decoding its position according to the encoding scheme, a continuous displacement prediction can be obtained, enabling precise continuous distance measurement.

Fig. S14 shows a set of experimental results using the same data splitting strategy as the first method: even-indexed classes serve as the main training set, a small portion of odd-indexed classes are mixed into the validation set, and the remaining odd-indexed classes are used as the test set. The results demonstrate good performance on the test set, achieving accurate continuous displacement prediction.

The prediction accuracy for even classes (Test A) is relatively high, while that for odd classes (Test B) is lower. There remains significant room for improving the choice of Gaussian hyperparameters and loss functions in this method.

Supplementary Note 6. Displacement detection and image reconstruction of optical fibers in different bending states

In semiconductor equipment displacement monitoring and minimally invasive surgeries, optical fiber probes are often required to pass through narrow gaps or cavities to reach the target location

for in-situ measurements, without the need to dismantle the mechanical casing. To address this, we conducted experiments on displacement detection using multi-mode fibers in different bending states to verify their resistance to bending.

Specifically, we changed the bending radius of the optical fiber probe at a distance of approximately 20 cm from the fiber-end-ball. Under three bending radii of 5 cm, 7.5 cm, and 10 cm, we collected speckle fields corresponding to 4000 grayscale patterns refreshed at displacement of 0 nm, 10 nm, 20 nm, 30 nm, and 40 nm. In total, 15 data sets were collected.

First, we verified that the optical fiber can detect displacement under different independent bending radii. We input the data from five displacements at the same bending radius into the VGG network for training. After shuffling the speckle dataset, 70% was used as the training set, 10% as the validation set, and 20% as the test set. Fig. S15a-c show the loss function curves, accuracy curves, and confusion matrices for the bending states of 5 cm, 7.5 cm, and 10 cm. It can be seen that under separate training, the displacement recognition accuracy for the three states can reach 99.95%, 99.98%, and 99.65%, respectively.

Next, we mixed the data from the three states and applied the joint learning method to attempt to achieve bending resistance. Specifically, the data corresponding to the same displacement value for three bending radii were mixed and shuffled, and then input into the VGG network for training. The resulting confusion matrix, shown in Fig. 4d of the manuscript, indicates that the displacement recognition accuracy reached 99.73%. This demonstrates that the system is capable of performing displacement recognition under different bending states.

To validate the system's imaging capabilities under different bending states, we conducted verification experiments. To simultaneously improve the bending robustness and maintain high-fidelity image reconstruction in an integrated multimode fiber imaging system, an additional network can be introduced to construct a "Bending-state–transmission-matrix library." In fact, a similar exhaustive pre-training approach has been applied in "light-field encoding" imaging (Ref. 27), enabling endoscopic examination in complex intracavitary environments—such as those with long-term presence of fluids, extended cavities, and temperature variations—in tissues like sheep small intestine and pig esophagus.

By employing two separate networks for bending state recognition and inverse transmission matrix optimization for imaging tasks, high-quality image reconstruction under different bending radii is achieved. Specifically, the five datasets corresponding to bending diameters from 14 cm to 22 cm were labeled with their respective bending radii and fed into a VGG classification network. The captured speckle data were randomly shuffled, with 70% used for training, 10% for validation, and 20% for testing. As shown in Fig. S16a, the bending radius identification results are presented, with the vertical axis representing the actual bending radius and the horizontal axis indicating the predicted bending radius. It can be observed that all data points align along the diagonal, indicating accurate identification.

Fig. S16b illustrates the architecture of the VGG classification network used for bending radius recognition. The input to the VGG bending state identification network is a diffuse speckle pattern compressed to a resolution of 128×128. The network first processes the input through a 3×3

convolutional layer, followed by a hyperbolic tangent (tanh) activation layer and a 2×2 max pooling layer. This structure is repeated sequentially, increasing the channel depth progressively from 32 to 256, while maintaining consistent kernel size, activation, and pooling operations throughout. The output is then flattened and fed into a fully connected layer with a size of 1×16384, using tanh as the activation function. Two subsequent fully connected layers gradually reduce the number of elements from 16384 to N, which corresponds to the number of bending sample categories. The model was trained for 50 epochs using the Adam optimizer with an initial learning rate of 1×10^{-3}, until convergence was achieved.

On the other hand, an inverse transmission matrix (ITM) optimization network was utilized to reconstruct images from data collected under different bending radii and to further extract the optimized inverse transmission matrices. These ITMs were labeled with their corresponding bending radii and integrated to construct a "Bending-state transmission matrix library." The specific testing workflow is illustrated in Fig. S16d. When an output speckle pattern captured under an unknown bending radius is acquired, it is first input into the VGG bending-state recognition network to identify the current bending state of the system. Based on the output of the VGG network, the corresponding inverse transmission matrix associated with the identified bending state is retrieved from the bending-state transmission matrix library and used for image reconstruction. The reconstruction results are shown in Fig. S16e.

The above results demonstrate the feasibility of the method using five sampling instances with bending applied at a single location and in one direction. However, it is expected that by increasing the number of sampling points, reducing the interval between bending diameters, and performing repeated sampling in multiple directions, this approach could theoretically enable image reconstruction under flexible bending of a single multimode fiber probe. Of course, handling such a large volume of data would require higher-speed acquisition efficiency and more powerful parallel computing capabilities.

Supplementary Note 7. Displacement detection and material recognition of different metal materials

To demonstrate the detection capability of the proposed multi-mode fiber system for different materials, we conducted displacement recognition experiments with plates made of red copper, brass, aluminum, and stainless steel, mounted on the nanometer displacement platform.

Specifically, we first cleaned the metal plates with alcohol and then fixed them onto the nano-electronic displacement stage. The plates were moved in the range of 0-400 nm with 100 nm intervals. At each position, 500 speckles were collected. To simulate potential vibrations during actual measurements, after the displacement stage reached 400 nm, we controlled it to move in reverse with the same 100 nm displacement interval, bringing the stage back to 0 nm. We then mixed the forward and backward displacement data for the same displacements and input the data into the VGG displacement recognition network for training. The displacement recognition accuracy for red copper, brass, aluminum, and stainless steel materials all reached 100%. Fig. S17a-c show the training loss function curves, recognition accuracy curves, and confusion matrices for red copper, brass, aluminum, and stainless steel.

Building on this, we mixed the data from the four materials for the same displacement value and applied the joint learning method to test the system's resistance to different materials. The resulting confusion matrix, shown in Fig. 4f of the manuscript, indicates that the displacement recognition accuracy remained at 100%. This proves that the system is capable of performing displacement detection across different materials. Fig. S17d shows the joint learning loss function curves, recognition accuracy curves, and confusion matrix for different metals.

During the experiment, we observed that the speckle field responded differently to different metal materials at the same displacement. This inspired us to explore the possibility of using the speckle field to identify the material of the detected target. Using the data collected above, we modified the mixing approach. Specifically, we mixed the data from different metal materials for the same displacement value and input the mixed data into the VGG network for training, attempting to perform material identification. Fig. S17e shows the training loss function curves, recognition accuracy curves, and confusion matrix for material identification. We found that the recognition accuracy also reached 100%, demonstrating that the multi-mode fiber speckle detection scheme has a high level of accuracy in identifying the material of the detected target. We believe this is of significant importance for optical fiber probes in searching for effective regions and recovering high-dimensional information.

Additionally, surface roughness is one of the key parameters characterizing the sample for displacement measurement. First, it should be noted that the method proposed in this manuscript, which employs diffuse speckle fields and a displacement recognition network, is not strongly dependent on the surface roughness of the sample. In other words, the method is applicable to samples with varying degrees of roughness, and within a wide range, surface roughness does not significantly affect the displacement recognition results. The following provides a more detailed analysis.

Except for certain mirror-like and engineered materials that undergo high-precision polishing techniques such as magnetorheological finishing or ion-beam figuring, the surface roughness of real-world objects is generally larger than 10 nm (the minimum displacement resolution presented in the manuscript). The materials used in our manuscript, such as wafers and metal plates, fall into this category.

To verify the surface roughness of the test samples, we measured a wafer and four additional metal plates (stainless steel, brass, aluminum, and copper) using a Park Systems atomic force microscope (AFM). Fig. S18a and S18b shows the selected sampling areas on the wafer surface and the measured height distribution within a 5 μm × 5 μm region. Fig. S18c-S18g further presents the two-dimensional height distributions and height histograms of the selected areas measured by AFM for all five material samples, illustrating the surface roughness.

It can be observed that within the selected sampling area of the wafer, the height variation, i.e., the peak-to-valley (PV) value, is approximately 50 nm. The other four metal plates (stainless steel, brass, aluminum, and copper) exhibit higher roughness, with PV values of 293 nm, 165.5 nm, 163 nm, and 150.5 nm, respectively. From their respective histograms and fitted curves, the height variations approximately follow a Gaussian distribution centered around zero.The above results are based on the height distributions measured within a 5 μm × 5 μm sampling region. In contrast,

the fiber probe used in the manuscript detects a larger area of approximately 400 μm × 400 μm, within which the samples are expected to exhibit even larger PV values and roughness. Even so, the proposed method, based on diffuse speckle fields and deep learning recognition, can still achieve nearly 100% recognition accuracy for the wafer at 10 nm resolution and for the metal materials at 100 nm resolution. This demonstrates the robustness and general applicability of our method to samples with varying surface roughness.

We would like to further emphasize that the method proposed in the manuscript is aimed at relative displacement measurement of the target object. It does not involve measuring the absolute height variations or absolute distances within the detection area (although this could be achieved through calibration if desired). For a rough surface, even if its height variations exceed the minimum measurement resolution, when the sample undergoes a displacement, the displacement at each point within the detection area is identical. Therefore, within a certain range, as long as the surface roughness does not significantly affect the reflected signal intensity, the height variations of the sample do not impact the measurement accuracy.

Supplementary Note 8. Compressed sampling of speckles for displacement detection and image reconstruction

To verify the feasibility of compress sampling in displacement detection, specifically, we extracted five regions with different sizes, named S1-S5, from the upper left 1/4 region of the entire speckle field at 508nm resolution, as shown in Fig. S19a and S19c. These five sets of compressive sampled speckles are separately fed into the VGG network for displacement recognition. 100% accuracy is achieved for all five sample-size partial speckles. To explore the relationship between sampling location and detection accuracy, the speckle images are further compressed into 5 different regions. Four corners are combined each time to form five sets of sampled regions named C1-C5. The sampling and processing process is shown in Fig. S19a and S19b. To exclude other factors interfering, the sampled data is compressed into images of 128×128 size. The accuracy remains at 100%. Fig. S19d illustrates the results of positioning with a resolution of 0.01 mm using the compressed sampled diffuse speckle field. Our system shows great robustness across all compressive sampled speckles. For different regions, the classification accuracy all converges at 100% after 5 epochs, while C1 sampling exhibits the highest convergence rate. The results perfectly match the conclusions in the manuscript. We also tested the compressed sampling results at the 508nm resolution. Fig. 5b and Fig. 5c are confusion matrices of the C1a and C5a sampling regions further compressed to 4×4 pixel size at 508 nm resolution in the range of 0-2032 nm, respectively. Average detection accuracy of 92% and 73%, respectively.

To further explore the compressed sampling limit for positioning, we divide the speckle into 20×20 small squares (see Fig. S19e). We utilized one part located on the diagonal for distance detection, labeled C1a'. The cropped C1a' is compressed to 4×4 pixels. Fig. S19f shows its positioning result. It is observed that, even after cropping and compressing the data to approximately 0.28‰ of mode number, a positioning accuracy of 93.1% is still achieved. Fig. S19g shows the positioning results of C10a', which is at the periphery. Compared to the center region, the positioning ability of the sampled speckles at the periphery is reduced. But it still has an accuracy exceeding 90%. Fig. S19h and Fig. S19i present the confusion matrix of C1a' region further compressed to 3×3 and 2×2

pixels, respectively. At this level of compression, the positioning accuracy decreases significantly. The C1a' region with 2×2 pixels has an accuracy of only 65.4%.

On the other hand, the number of pixels in the speckle image is directly related to the number of layers and parameters of the neural network and the number of elements to be optimized in the inverse transfer matrix. Consequently, employing small-size speckle fields imaging contributes to enhancing the computational speed of image reconstruction and reducing hardware configuration requirements.

To find highly informative sampling regions with little computed pixels, different sampling methods are experimented with to explore the image reconstruction capabilities of compressive sampled speckles. The size of the sampled speckle and the sampling position are varied to find the optimal sampling approach. Specifically, we extracted five sets of compressive sampled speckles at S1-S5 regions(see Fig. S19a and S19c) are separately fed into the inverse transmission matrix optimization network for image reconstruction. Fig. S20a demonstrates the image reconstruction results for the five regions of speckles from S1-S5. It is obvious that the reconstruction quality improves as the speckle range increases. However, this relationship is not linear. As can be seen from the blue curve in Fig. S20c, the growth rate of structural similarity slows down as the area increases. A reconstruction result with structural similarity (SSIM) of more than 0.45 can be achieved using the region S3 with only 9% of the full area of the speckle field.

On this basis, the relationship between sampling location and imaging quality is explored. We extract the C1-C5 regions(see Fig. S19a and S19b). To exclude other factors interfering, the sampled data is recompressed into images of 128×128 size. The reconstruction results are shown in Fig. S20b. From the reconstruction results and the red curve in Fig. S20c, it can be seen that there is a discrepancy in the information-bearing density of the speckle region at different locations. The SSIM of the reconstructed image increases and then decreases with increasing radius, and reaches the maximum near the C2-C3 region. In order to verify the credibility of the results, we conducted repeated experiments. The results of repeated experiments form the fluctuations in the error band around the red solid line in Fig. S20c. In addition, we fed single 1/4 corners, C1a-C5a, into the network for training. The results obtained are shown in the yellow curve in Fig. S20c, demonstrating a similar trend of change.

From the Fig. S20, there is a discrepancy in the information-bearing density of the speckle region at different locations. The SSIM of the reconstructed image increases and then decreases with increasing radius, and reaches the maximum near the C2-C3 region. These results are due to a combination of factors such as speckle size, number of mode orders contained, and signal light intensity. The closer to the center of the speckle field, the scattering spots in the speckle have a larger size. And less information is contained in sampled regions with the same sample size. At the same time, the low-order modes are mainly concentrated at the center region, while the high-order modes that respond to the image details are mainly distributed at the periphery. Conversely, when the sampling region is located at a radius that is too large, the intensity of speckles diminishes. The total number of combined modes is reduced, and the signal-to-noise ratio is low.

To sum up, we have realized the recovery of the whole image using partial speckles, and further explored the relationship between sampled speckle location and information capacity. This has

two advantages: Firstly, if we keep the same number of sampled pixels as the original, we can increase the spatial sampling rate of the speckle, so as to obtain more detailed scattering information, which can help to improve the quality of image reproduction. Secondly, the scattering sampling can compress the data significantly, which can save the computational cost of the network and improve the computational speed. The saved computational cost can be used to improve the resolution of the reconstructed image.

Supplementary Note 9. Integrated System Architecture

To reduce the probe size and improve the portability and implantability of the system, we integrated the optical path structure of the multi-mode fiber displacement detection system. Specifically, we used a side-coupler to integrate the illumination path and detection path. Illumination light is coupled into the multi-mode fiber probe through an illumination fiber, with most of the illumination light field propagating through the cladding of the multi-mode fiber. To homogenize the collimated illumination field, we fused a fiber-end-ball at the fiber probe's detection facet. The fiber-end-ball serves as a substitute for the traditional lens set, further reducing the probe size. After the light field is output from the fiber-end-ball, it illuminates the target and is reflected. The light field carrying displacement information is then received again by the fiber-end-ball, propagated back through the multi-mode fiber, and finally detected by the CMOS sensor. The entire system does not contain any discrete optical components, achieving a highly integrated and all-fiber structure. The structure of the whole system is shown in Fig. S21.

9.1 Detection through the slit

In order to verify the detection ability of the fiber probe in a narrow space, we inserted the fiber into a capillary glass tube and detected the wafer. As shown in Fig. S22, the fiber optic probe can be easily inserted into a glass tube cavity channel with an inner diameter of 1.1 mm and the output speckle is obtained normally, which proves the detecting ability of the system in a narrow slit or cavity.

9.2 Lens-free displacement detecting and imaging

Existing speckle-based image transmission systems within step-index MMF waveguides rely on the fiber proximal facet projected on a camera. Reconstruction algorithms are realized by demodulating the output focused speckle field on the facet. However, in the above we have demonstrated that it is possible to use diffuse scattering speckle fields or even partial scattering fields for displacement detection and reconstruction of the full field of view of the input image, even though the preprocessing crops and loses some of the speckle information. This is feasible because when the light field passes through a strong scattering medium, the randomness and anisotropy of the medium perturbate the light in an irregular manner, making not all of the output speckle information essentially needed to reconstruct the input image at the distal end of the MMF. In fact, when the length of a MMF is much larger than the scattering distance and the radius of the fiber cross-section, the light field at any point of the output speckle field is related to the light field intensity information(image) and phase information(displacement information) at all points of the input field.

$$I(x,y) = \left| \iint_{-\infty}^{\infty} t(x_0, y_0, x, y) E(x_0, y_0)\, dx_0 dy_0 \right|^2 \tag{S26}$$

where $I(x,y)$ is the output scattered field intensity distribution, $E(x_0, y_0)$ is the input plane light field distribution, $t(x_0, y_0, x, y)$ is the superposition factor of the light field $E(x_0, y_0)$ at (x_0, y_0) of the input plane on the scattering field at (x, y) of the output plane.

The mapping relationship between input and output can be found by solving the superposition factor. Therefore, it is not necessary to use all the output speckle field parameters, but it is generally required that the number of speckle pixels is greater than the total number of pixels of the input image. This characteristic of speckles similar to holographic plates provides a theoretical basis for lensless multimode fiber displacement detection and image reconstruction.

9.3 Optimization of the air layer thickness

Due to the removal of all lenses, there is an air layer between the fiber output facet and the CMOS sensing surface. The air layer thickness is a critical factor affecting the accuracy of reconstructed images. When light propagates through an air layer, it is multiplied by a transfer function in the frequency domain, resulting in changes to the transmission matrix, thereby affecting the imaging results. On the other hand, this thickness also affects the area occupied by the overall diffuse speckle and the average size of the scattering spot. If the thickness is too large, the received higher-order mode speckles intensity is weakened and the signal-to-noise ratio is reduced; if the thickness is too small, the limitation of the sensor size averages the speckles intensity and causes the loss of effective information[9]. The thickness of the air layer in the experiment is about 4.8 mm.

Different air layer thicknesses are tested separately. As the thickness decreases, the intensity of the speckles received on the detector increases and the diffuse speckles gradually converge. The image reconstruction results also showed an upward trend. However, if the thickness is further reduced, the scattering spot size and the spacing between neighboring scattering spots will further decrease. When the size of the scattering spot irradiating onto the sensor is smaller than the sensor pixel size, this means that the intensity received by a single sensor pixel will be the average of multiple spots. We consider this to be a degradation of image quality.

Specifically, to obtain the best experimental results, the detected distance between the DMD and the fiber-end-ball is fixed while changing the thickness between the fiber facet and the sensor. When reducing the thickness, we appropriately lowered the laser power to ensure that the maximum grey value at the center of the speckle could reach 200 and the sensor will not be overexposed. The exposure time is uniformly set to 50,000 μs. Due to the limitation of the protective window in front of the sensor, the minimum thickness in the experiment is 4.8mm. The thickness is changed from 4.8mm to 7.8mm with an interval of 1mm, and the corresponding laser powers are 5mW, 6mW, 7.5mW, and 8mW. The results are shown in Fig. S23. It can be seen from the results that as the air thickness increases, the imaging quality deteriorates. We also performed displacement recognition experiments using the same wafer pattern at four different distances from the CMOS sensor: 4.8 mm, 5.8 mm, 6.8 mm, and 7.8 mm. At each distance, we collected 5 sets of diffuse speckle patterns at 10 nm displacement intervals, with each set containing 1000 speckle

fields, resulting in a total of 20 sets of data. Each dataset corresponding to a specific distance was independently used for training and testing, yielding the four confusion matrices shown in Fig. S23.The displacement measurement results were not affected by the measurement distance. However, if the thickness is reduced to a certain value, the imaging quality may also be degraded due to the relationship between the speckle size and the sensor pixel size.

For the multimode fiber used in this experiment, the diameter of most of the scattering spots at the output facet is about 0.5-4.5 μm. The pixel size of the sensor is 3.45 μm. Therefore, in the future, if sensors are integrated onto the multimode fiber facet without an air layer, a sensing chip with much smaller pixels will be necessary.

9.4 Optimization of the detection angle

Ideally, the fiber probe should be aligned vertically with respect to the sample surface. However, in practical experiments and real-world usage, strict vertical alignment is difficult to achieve. We would like to point out that even if the fiber is not perfectly perpendicular to the sample surface, as long as the displacement direction remains parallel to the fiber axis, the field of view at different heights can still remain consistent. On the other hand, if the displacement direction forms an angle with the fiber, the field of view and the detected speckle pattern will change with height. Nevertheless, as long as this angle remains constant, accurate displacement recognition is still achievable, as demonstrated in Fig. 3e of the manuscript.

To address your concern regarding the fiber-to-sample tilt, we performed comparative experiments to investigate its effect on displacement recognition. It should be noted that most of our samples exhibit specular reflection, meaning that there exists a specific reflection angle at which the returned signal is significantly stronger within a certain range. In the comparative experiment, the wafer was placed on a rotation stage, and the reflected light received by the camera was observed. The midpoint of the high-intensity reflection angle range was taken as 0°. The rotation stage was then sequentially rotated, and the diffuse speckle fields captured by the camera were recorded at ±0.5°, ±1.5°, ±2.5°, ±3.5°, and ±4.5°, while keeping the sample-to-fiber distance constant, as shown in Fig. S24a. From the figure, it can be seen that as the tilt angle deviates further from 0°, the intensity of the captured returned light gradually decreases, resulting in lower speckle contrast and reduced signal-to-noise ratio. When the tilt angle exceeds 5°, the camera barely receives any returned signal, making effective acquisition impossible.

Furthermore, to directly evaluate the impact of tilt angle on displacement recognition, we acquired five sampling points at each angle with a displacement resolution of 10 nm, and for each sampling point, 1000 diffuse speckle fields were collected. These data were then input into the displacement recognition network for training. The resulting confusion matrices for each angle are shown in Fig. S24b. From the figure, it can be seen that within the range of –1.5° to 0.5°, displacement recognition achieves 100% accuracy. When the probe tilt exceeds this range, the recognition accuracy drops sharply, rendering the results completely unreliable. Therefore, we conclude that the system can achieve high-accuracy displacement recognition within an effective angular range of approximately ±2°. The fiber does not need to be perfectly perpendicular to the sample surface, but it should remain within this effective angular range.

In practice, to ensure effective data acquisition, before each experimental session, the tilt angle between the sample and fiber is adjusted in real time by observing the intensity of the diffuse speckle fields captured by the output camera. This procedure ensures that the acquired data maintain sufficient signal-to-noise ratio for reliable displacement recognition.

9.5 Comparison of the proposed lensless system with the conventional multimode system

In conventional optical imaging systems, lens-like discrete optical elements are present between the imaged object and the detector device. We experimentally compared the proposed system with the conventional lensed system under the same fiber type and system configuration.

For displacement detection ability comparison, the acquired facet speckle images and the diffuse speckle field are put into the VGG network with the same parameters. Specifically, we input 40,000 images from the grayscale dataset at five different displacements into the two systems, each displacement corresponding to 8,000 images. The networks are trained for 50 epochs. The acquired facet speckle images and the diffuse speckle field are put into the VGG network with the same parameters. The loss accuracy curves for the training and validation sets are shown in Fig. S25b. The average positioning accuracy of the lens system is 82% and that of the lensless system reaches 100%. The normalized confusion matrix is shown in Fig. S25a. We consider that the superiority of the lensless system in displacement detection is brought by the increased differentiation of the speckles at different displacements. In the simulation results of Fig. 2, the displacement of the target mainly leads to the change in the percentage of high-order modes. These high-order modes with large divergence angles occupy a larger distribution area in the diffuse speckle field instead of being confined within the fiber core.

For image reconstruction ability comparison, the two systems acquire the same 8000 sets of data, including 7200 sets for training, 400 sets for validation, 400 sets for test. The two systems underwent 100 iterations using identical hardware and network configurations. The image reconstruction results of their test sets are shown in Fig. S25c. After the same 100 iterations, the SSIM of the reconstructed images of the test set for the lens system is 0.583, and that of the lensless system is 0.623. For comparison, the top and bottom recovered images of each set of pictures also show that the image reconstruction results of the lensless system have reached or even outperformed those of the lens system. This may be attributed to the cropping method of the lensed system results in a black background in the four corners of the speckle field that lacks information, as well as the enhanced stability of the system achieved by the removal of the discrete lens.

Supplementary Note 10. Reconstruction from Frequency Spectrum of the Diffuse Speckle Field

Whether the imaging system uses a multi-core fiber bundle or a multi-mode fiber, an objective lens is placed at the output end of the fiber to receive the output light field. Conventional lenses can eliminate the quadratic phase factor of Fresnel diffraction, and the modulation can be viewed as a Fourier transform[10], which is equivalent to taking a Fourier transform. For further comparison with the lens system, we performed a Fourier transform of the output diffuse speckle field. The frequency domain magnitude field is fed into the inverse transmission matrix optimization network for training. Fig. S26 above shows the corresponding frequency domain magnitude fields of the

diffuse speckle fields and the reconstructed images from them. The average structural similarity of the results is 0.42. Image reconstruction is available, but imaging quality is limited. This is due to the absence of the phase field and the loss of spectral information caused by the cropping of the diffuse speckle field.

Supplementary Note 11. Optimizing imaging quality with the Displacement-Inverse Transmission Matrix Library（DITML）

The lensless system can generate reconstruction results for different displacements. In fact, targets with different displacements should correspond to different transmission matrixes. And for diffuse speckles with a large degree of variation at different displacements, joint learning[10] is no longer applicable. In practical applications such as endoscopy or industrial inspection, it is difficult to ensure that the transmission matrix for practical use matches the test transmission matrix. The proposed system, leveraging positioning capabilities with an accuracy approaching 100%, selects the most suitable transmission matrix for reconstruction, significantly enhancing the quality of imaging. The overall detection flow of the lensless system is shown in Fig. S27. First, the objects are placed at different displacements, and the system is sequentially trained using the inverse transmission matrix network to obtain the corresponding transmission matrices at different displacements to form a matrix library. Next, when the system actually detects an object, the captured diffuse speckle is first put into the VGG network for positioning. After obtaining the specific displacement, the corresponding transmission matrix is called from the labeled DITML for imaging.

Supplementary Note 12. 3D micro cross-section reconstruction by diffuse speckles

Despite breakthroughs that have been made in the transmission of flat images via a single MMF, for practical applications such as ultra-thin endoscopy and industrial inspection, the tissue or mechanical structure being measured is often uneven.

The left side of Fig. S28a illustrates the displacement detection by the MMF probe. By feeding the captured diffuse speckle into the VGG network and the inverse transmission matrix optimization network, respectively, the system not only senses where it is located, but also reconstructs the image behind the other end of the MMF. Fig. S28b shows the detection and reconstruction results of the images at five different displacements with 0.5mm resolution. After no more than 10 epochs, the positioning accuracy can be above to 99%. Displacement measurements with a resolution of up to 10 nm.

Incorporating positioning capabilities into the single multimode fiber imaging system can significantly enhance its functionality and applicability. The high spatial resolution not only improves image reconstruction quality, but also facilitates micro 3D structures imaging. Micro 3D structure reconstruction in deep and narrow environments requires not only precision positioning, but also a robust performance of image reconstruction throughout the overall detection depth of field. As shown on the right side of Fig. S28a, we intercept the isometric cross-sectional view of the 3D heart-shaped structure and load them onto the DMD, placing them sequentially at different displacements. The output diffuse speckles corresponding to different cross-sections are captured. And the contours at different positions of the 3D micro-structure are realized to reconstruct. Fig.

S28c illustrates the reconstruction results of the microscale 3D heart-shaped structure at 10 μm intervals for the 5 cross-sectional views from 0-40 μm. The patterns of speckle fields produced by a micro 3D object vary according to the distance between its surface and the probe, which is the fiber-end-ball in our setting. For every speckle field generated from an unknown position, the displacement can be recognized through VGG network. And then the image can be reconstructed by retrieving the corresponding transmission matrix. Therefore, the process of capturing the speckle fields, positioning by VGG network, retrieving the transmission matrixes, and reconstructing the distorted cross-section views can iterate many times until a micro 3D structure of the entire detection area is reconstructed.

The system was experimented with for the detection and reconstruction of simple 3D structures such as spheres, cylinders, cones, tetrahedrons, rings, hearts, and stars. The micro 3D cross section projected on the DMD as the input image changes with the distance between the probe and the micro 3D object, simulating the process of scanning through a micro 3D object in reality. The input cross-section images and the reconstructed images are given in Fig. S29. Our system steadily presents good detection and imaging results on these different micro 3D structures. Further reducing the interval distance and increasing the number of samplings, the proposed system has the potential for three-dimensional reconstruction.

Due to limitations in the experimental platform and materials, this is only a preliminary attempt at the feasibility of micro 3D imaging. Nevertheless, such excellent reconstruction results convince us that the proposed lensless speckle imaging method has a promising application in the field of micro 3D structure reconstruction in narrow and extreme environments.

Supplementary Figures

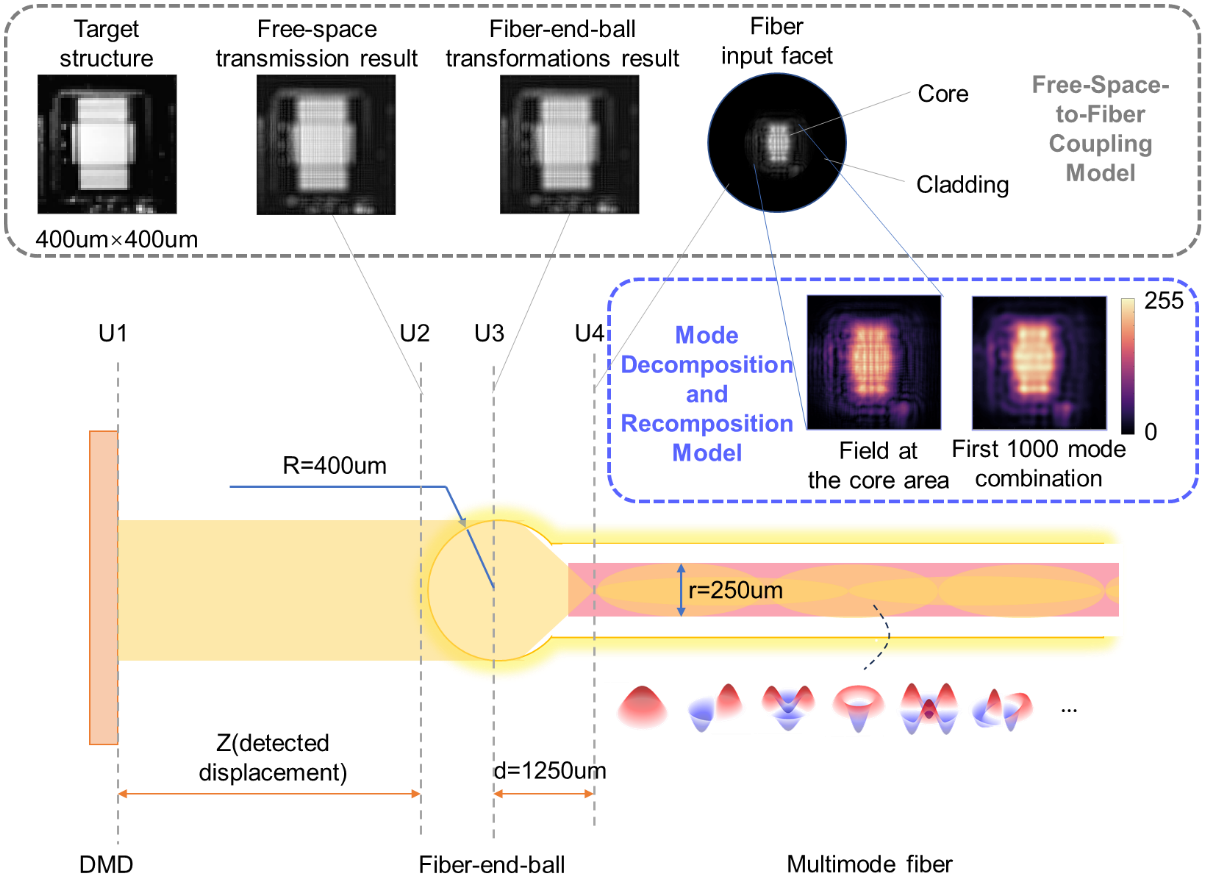

Fig. S1. Simulation architecture for optical field evolution of multimode fiber probes. Simulated target structure is transformed into a large number of transverse modes by free-space transmission, end-ball transfer, and mode decomposition. U_1: Simulated target structure by DMD modulation; U_2: Light field after free-space transmission; U_3: Light field after fiber-end-ball transformation; U_4: Light field at the input facet of the MMF. The Fiber input facet obtained from the Free-Space-to-Fiber Coupling Model is used for mode decomposition and recomposition.

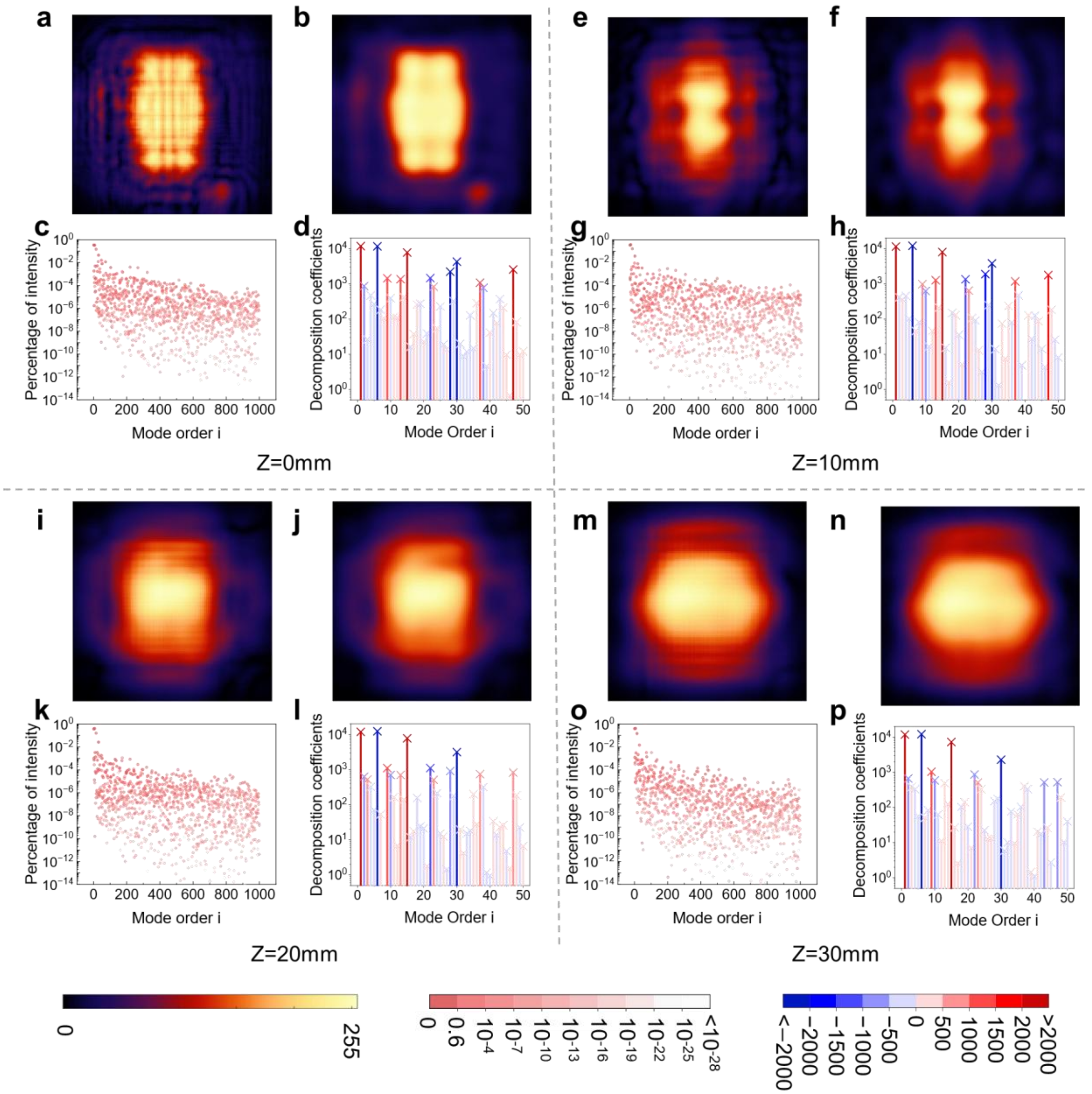

Fig. S2. Simulation results of the physical model at different displacements (**z=0mm, z=10mm, z=20mm, z=30mm**). **a, e, i, m,** Light Field at input facet(U4); **b, f, j, n,** Recombination of the first 1000 patterns; **c, g, k, o,** Percentage of the intensity of different modes; **d, h, l, p,** Decomposition coefficients of different modes.

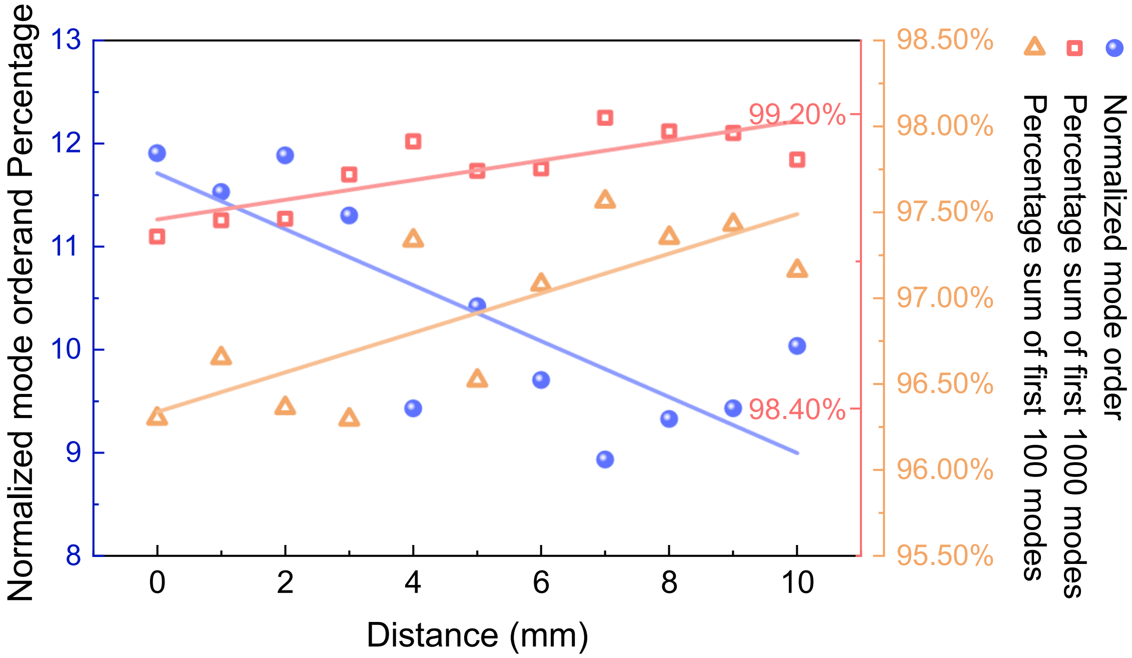

Fig. S3. Variation in normalized mode order, percentage sum of first 100 modes, and percentage sum of first 1000 modes with displacement distance under a wide-range low-resolution measurement (0–10 mm).

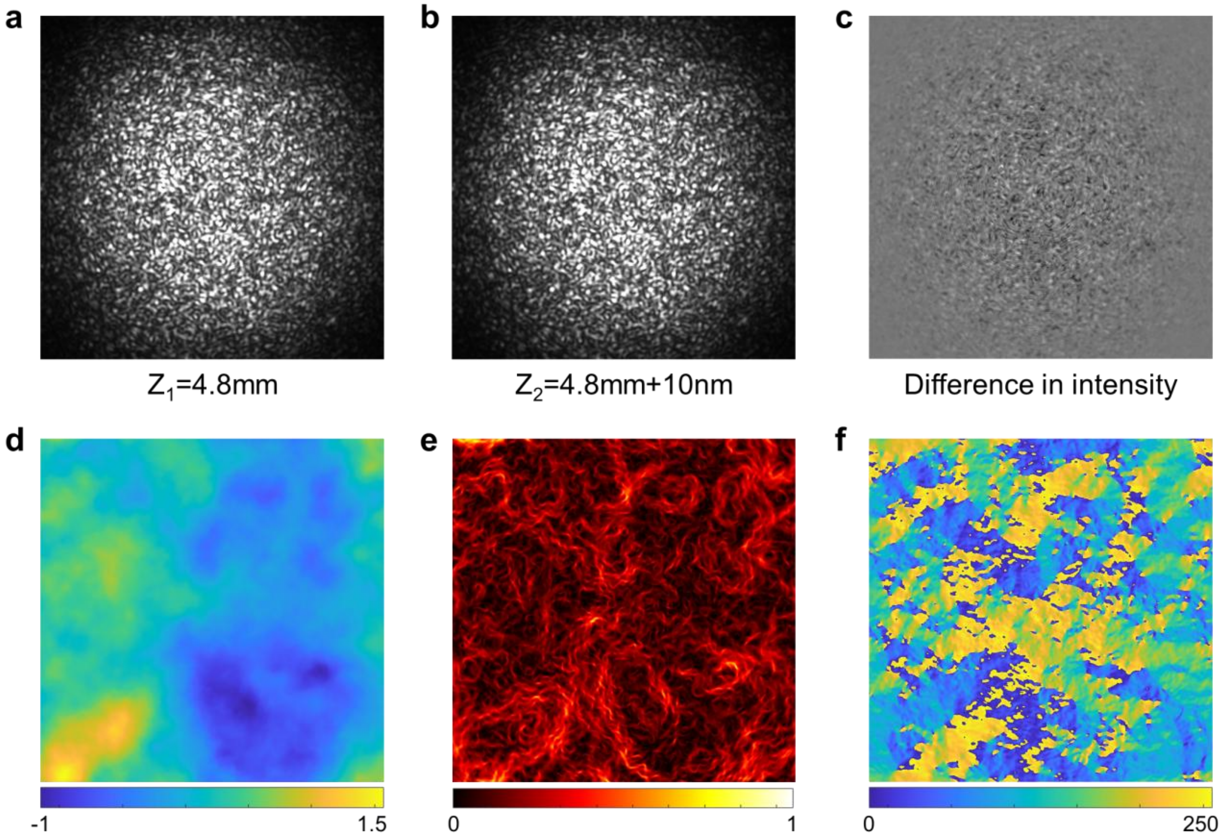

Fig. S4. Superoscillation in speckle patterns of multimode fiber. a, b, The speckle patterns captured at 4.8 mm and 4.8mm+10nm from the fiber end face; **c,** The differential result of the two speckle patterns; **d,** The phase distribution of the speckle field near 4.8 mm; **e, f,** The magnitude and direction distribution of the phase field gradient.

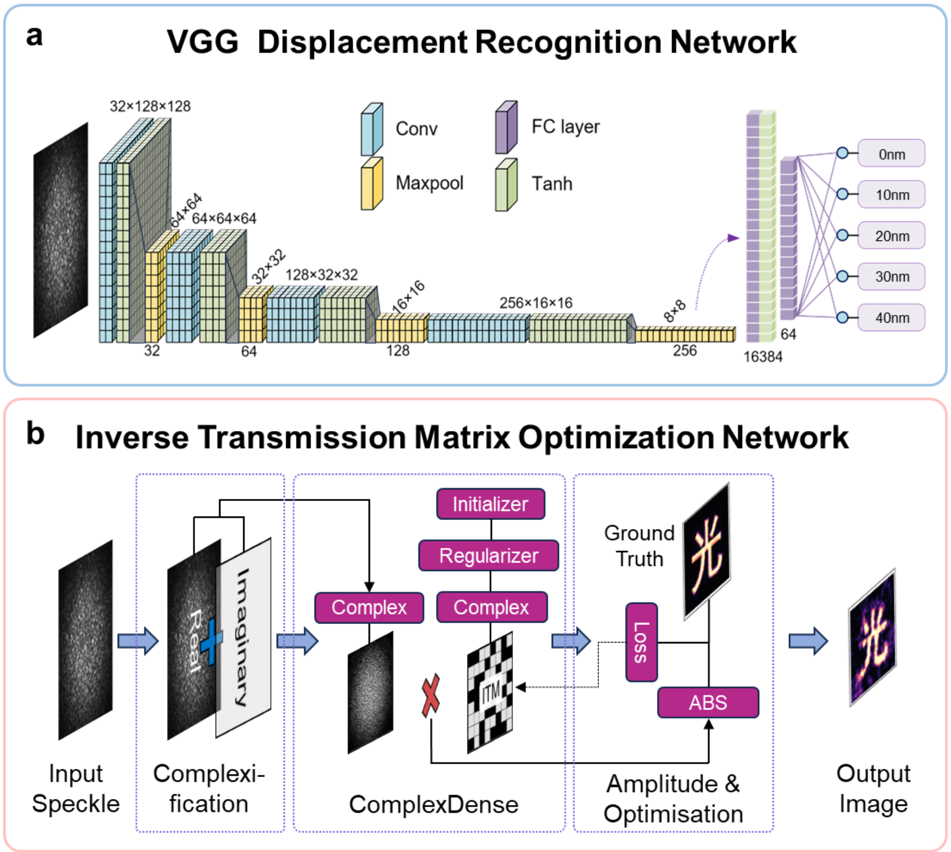

Fig. S5. Architecture of Neural Network. a, The architecture of VGG displacement recognition network. **b,** The architecture of inverse transmission matrix optimization network for image reconstruction.

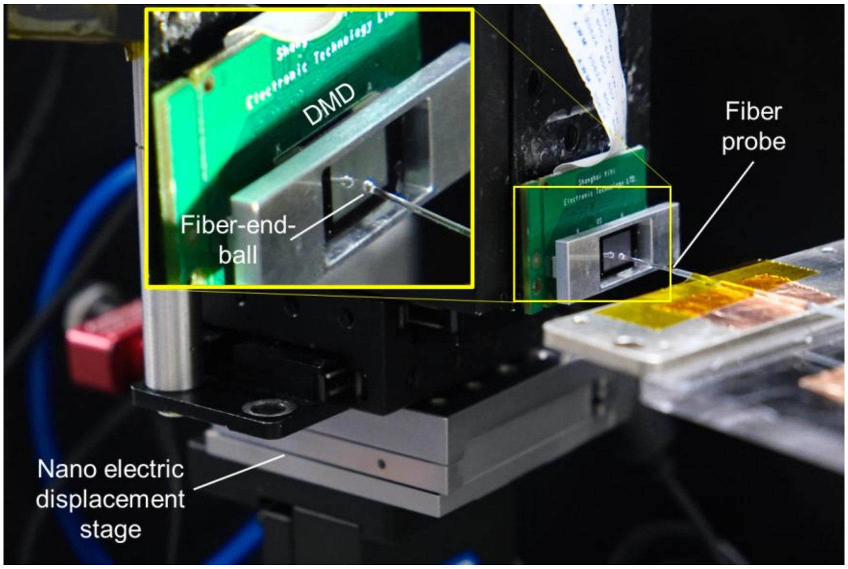

Fig. S6. Experimental setup for high-precision displacement detection experiments.

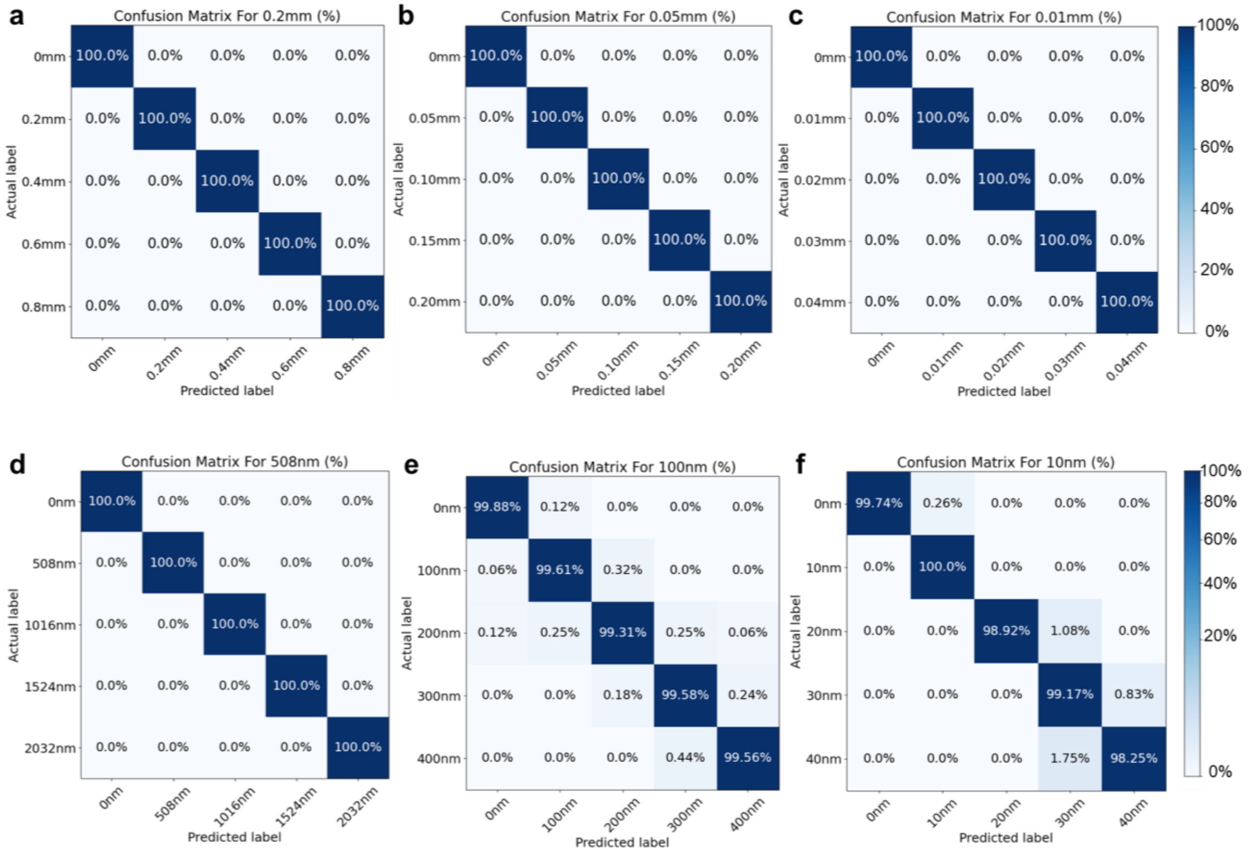

Fig. S7. Detection results of the proposed system at different displacement resolutions. **a,** Confusion matrix for displacement detection by multimode fiber system at 0.2 mm resolution. **b,** Confusion matrix for displacement detection by multimode fiber system at 0.05 mm resolution. **c,** Confusion matrix for displacement detection by multimode fiber system at 0.01 mm resolution. **d,** Confusion matrix for displacement detection by multimode fiber system at 508 nm resolution. **e,** Confusion matrix for displacement detection by multimode fiber system at 100nm resolution. **f,** Confusion matrix for displacement detection by multimode fiber system at 10 nm resolution.

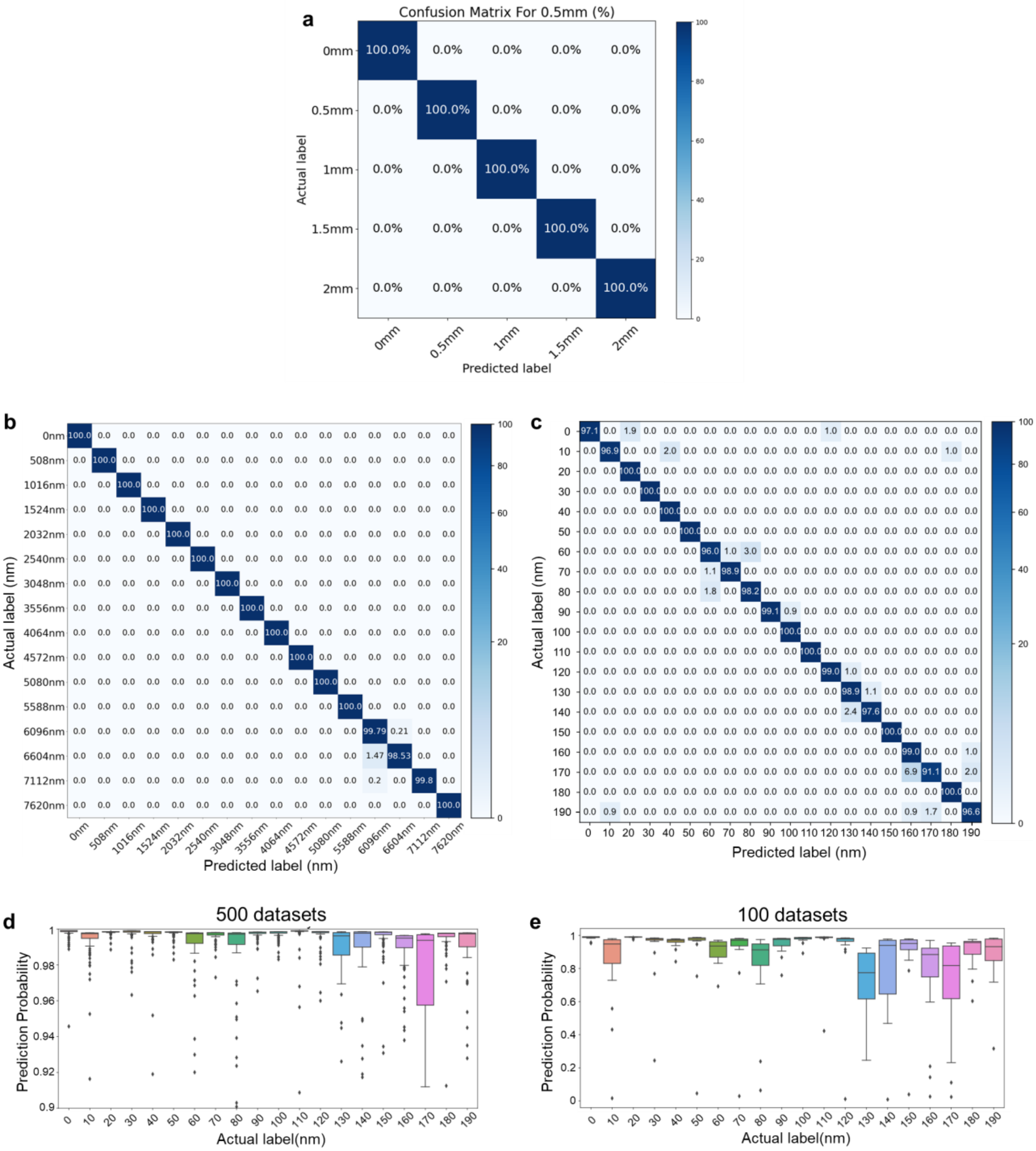

Fig. S8. Experimental results of system range. a, Confusion matrix of multimode fiber system for displacement detection in 0-2 mm range. **b,** Displacement detection confusion matrix for the multi-mode fiber system with 16 sets of sampling points at 508 nm resolution. **c,** Displacement detection confusion matrix for the multi-mode fiber system with 20 sets of sampling points at 10 nm resolution. **d,** Box plots of the probability distribution of correct recognition on each displacement for the network trained with 500 datasets per displacement. **e,** Box plots of the probability distribution of correct recognition on each displacement for the network trained with 100 datasets per displacement.

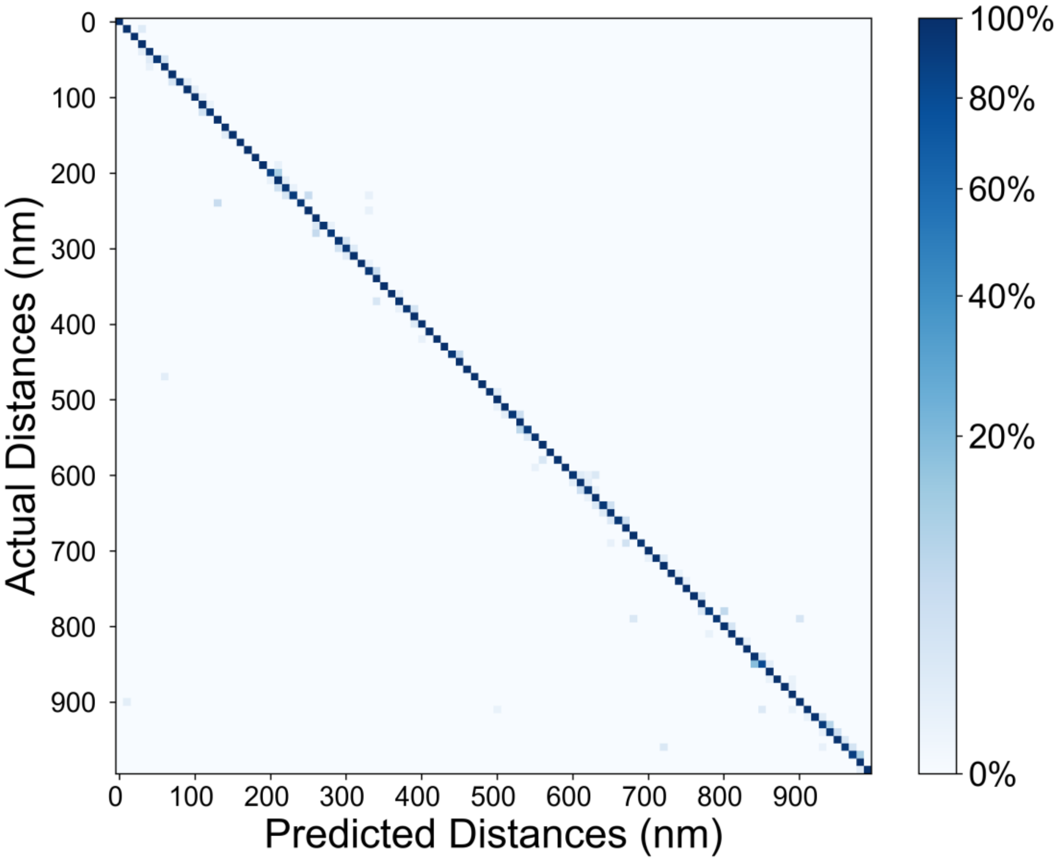

Fig. S9 Confusion Matrix for Displacement Recognition at 10nm High Resolution with a Wide Measurement Range of 0–990 nm (100 Sampling Points).

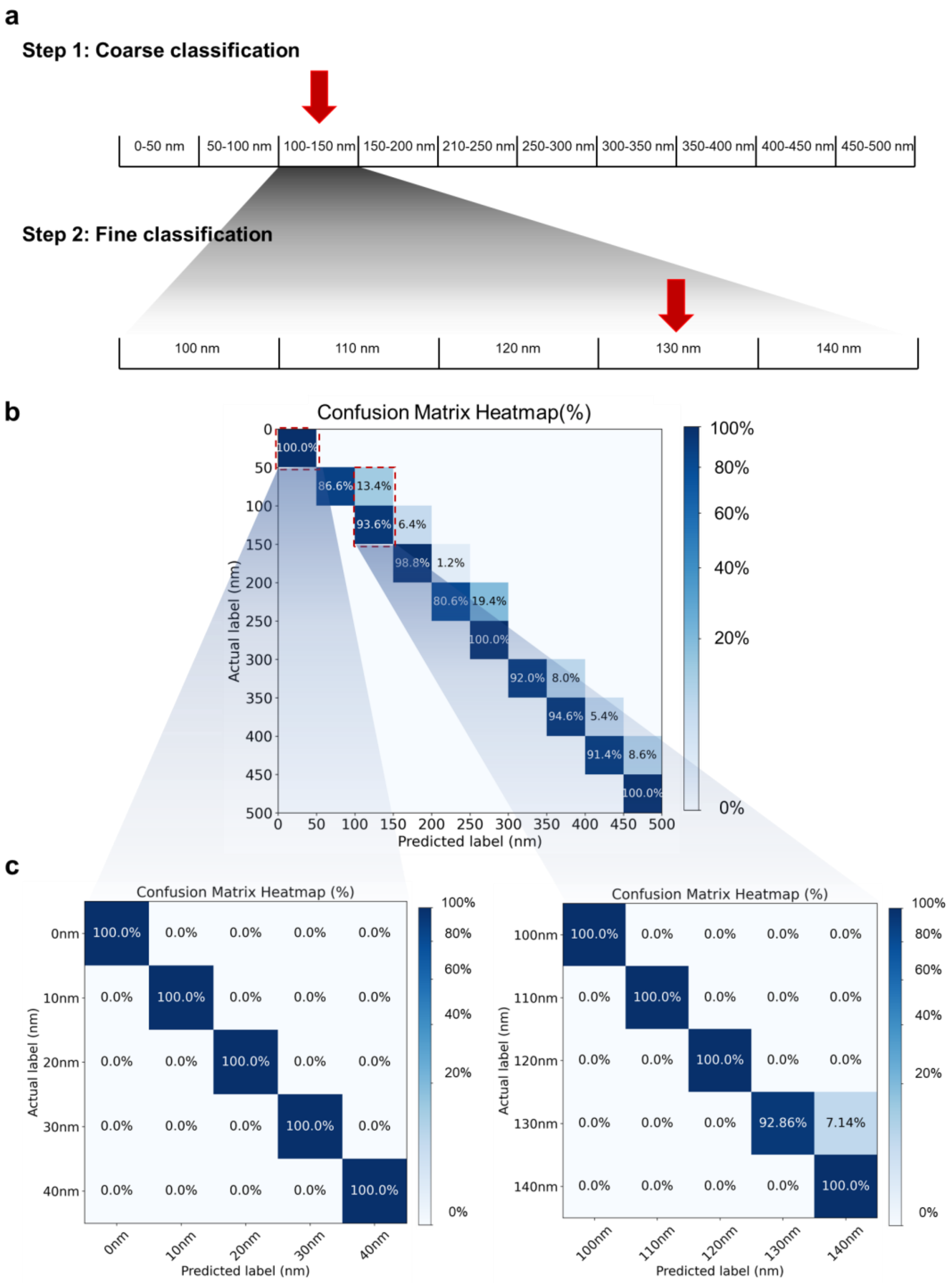

Fig. S10. Multi-interval partitioned recognition strategy. a, Overall process concept of multi-interval partitioned recognition. **b,** Hierarchical classification strategy for long-range and high-resolution displacement recognition. Confusion matrix of the coarse classification, where every 5 adjacent displacement classes are grouped into one super-class. The model effectively distinguishes between these coarse intervals, demonstrating the feasibility of using hierarchical classification to extend the measurable displacement range. **c,** Confusion matrices from two representative fine classification tasks within selected intervals. In both cases, the model accurately resolves fine-grained displacement variations, indicating stable performance and high spatial resolution in local regions.

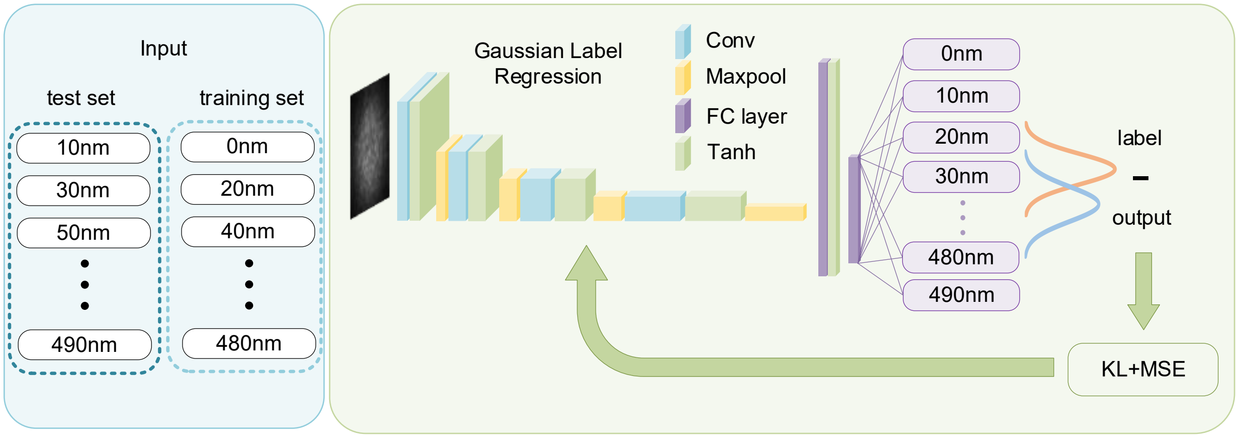

Fig. S11. Illustration of the Gaussian soft-label network. The network employs Gaussian labels centered at the true displacement, assigning decreasing probabilities to neighboring classes instead of conventional one-hot labels. A combined KL divergence and MSE loss function is used during training. Even-indexed classes are marked as the training set, while odd-indexed classes serve as the test set.

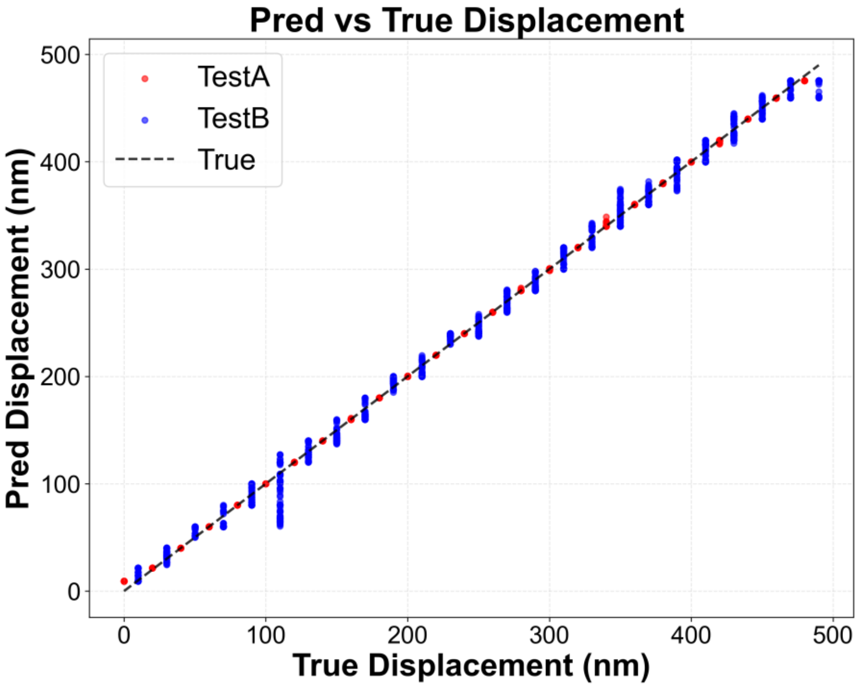

Fig. S12. Displacement recognition results of the Gaussian soft-label network. TestA is remaining even-indexed classes which accuracy is relatively high and TestB is odd-indexed **classes which accuracy is lower.**

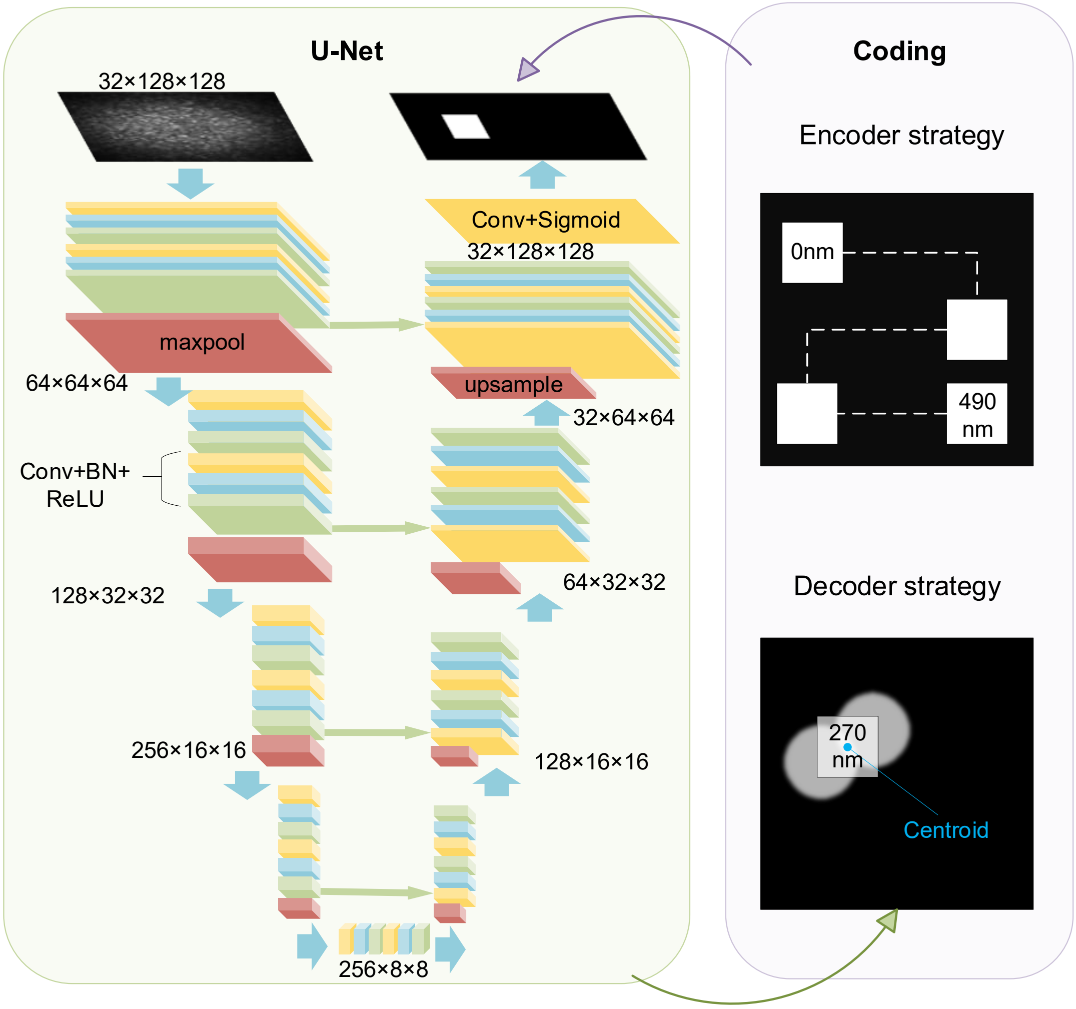

Fig. S13. Schematic diagram of the U-Net architecture. The network repeatedly uses two consecutive blocks of "Convolution + Batch Normalization (BN) + ReLU", which is a typical structure in U-Net. Skip connections (indicated by green arrows in the left panel) are incorporated to better retain features across different levels. The right panel illustrates the S-shaped encoding scheme of the input speckle patterns and the decoding process based on the centroid position of the predicted output pattern.

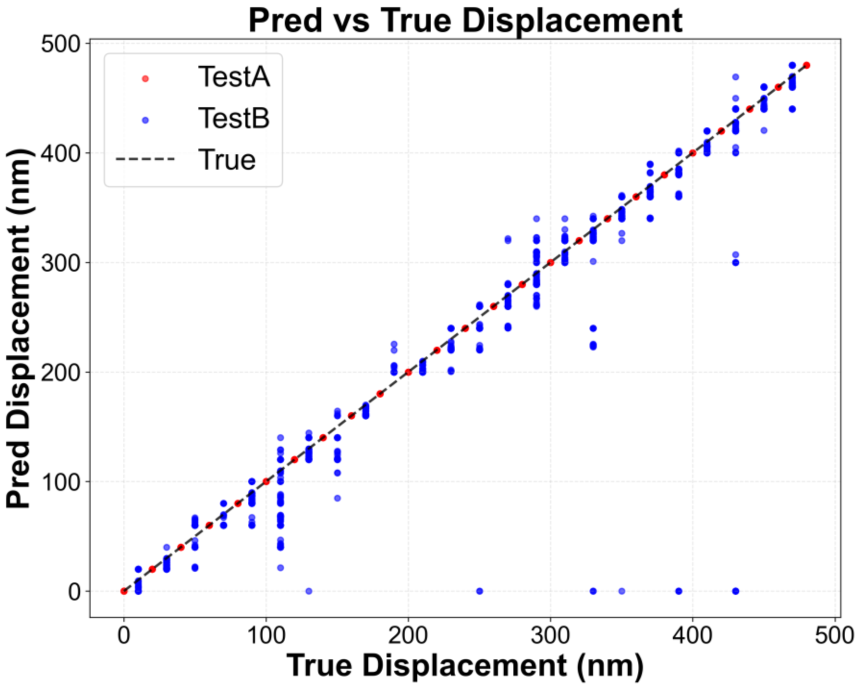

Fig. S14. Displacement recognition results of the U-Net network. TestA is remaining even-indexed classes which accuracy is relatively high and TestB is odd-indexed **classes which accuracy is lower.**

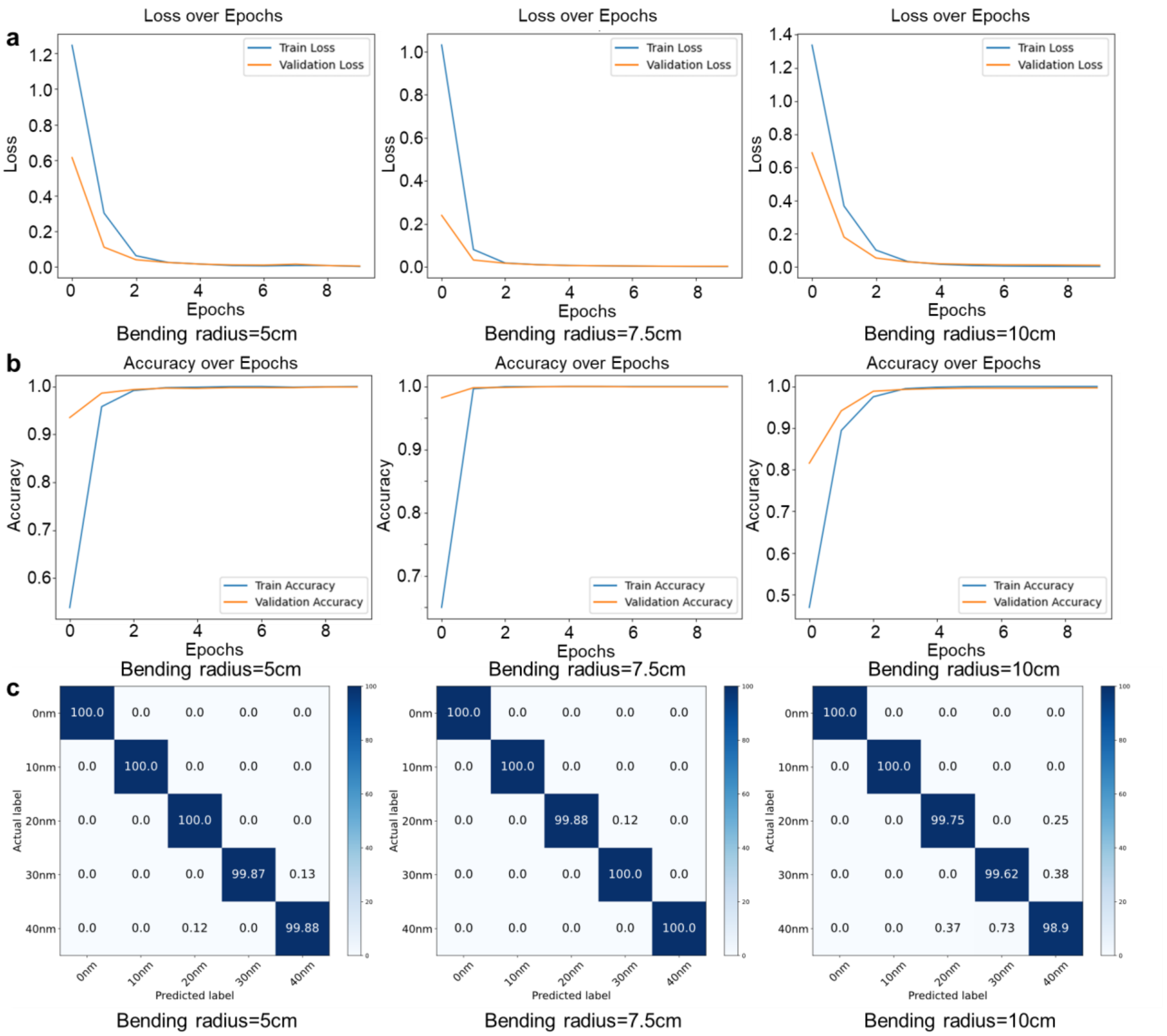

Fig. S15. Experimental results of multimode fiber system under different bending states. a, The loss function curves for the bending states of 5 cm, 7.5 cm, and 10 cm. **b,** Accuracy curves for the bending states of 5 cm, 7.5 cm, and 10 cm. **c,** Confusion matrices for the bending states of 5 cm, 7.5 cm, and 10 cm.

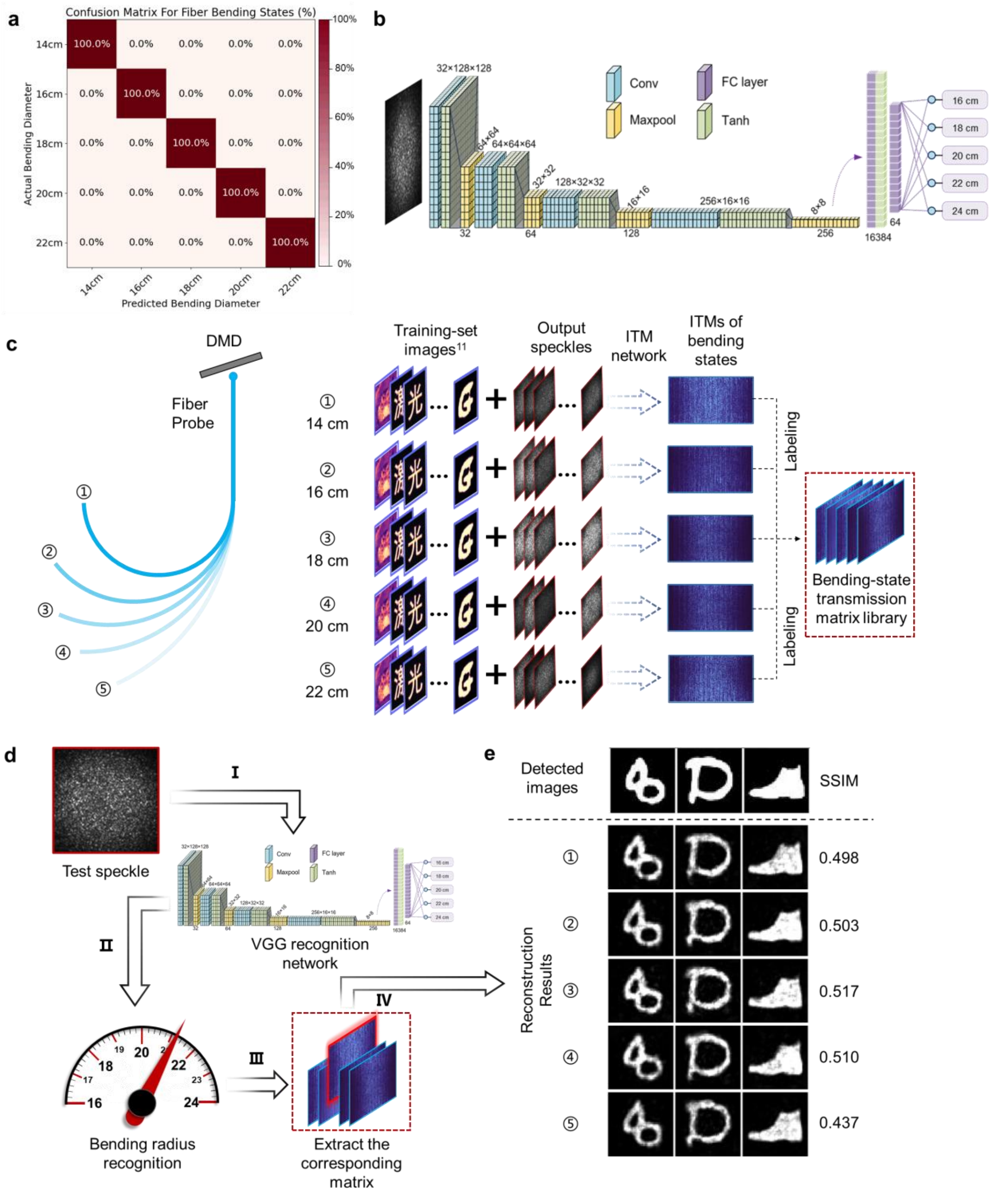

Fig. S16. Imaging results of multimode fiber system under different bending states. a, Confusion matrix for bending state recognition. **b,** The architecture of VGG bending state recognition network. **c,** Computing the ITM for a corresponding bending state by acquiring multiple speckle images under different bending conditions. The target images are grayscale natural pictures[11], homemade Chinese characters and patterns.**d,** Implementing a high-quality bend-resistant imaging workflow using a bending state recognition network and invoking the "Bending state-transmission matrix library." **e,** Image reconstruction results.

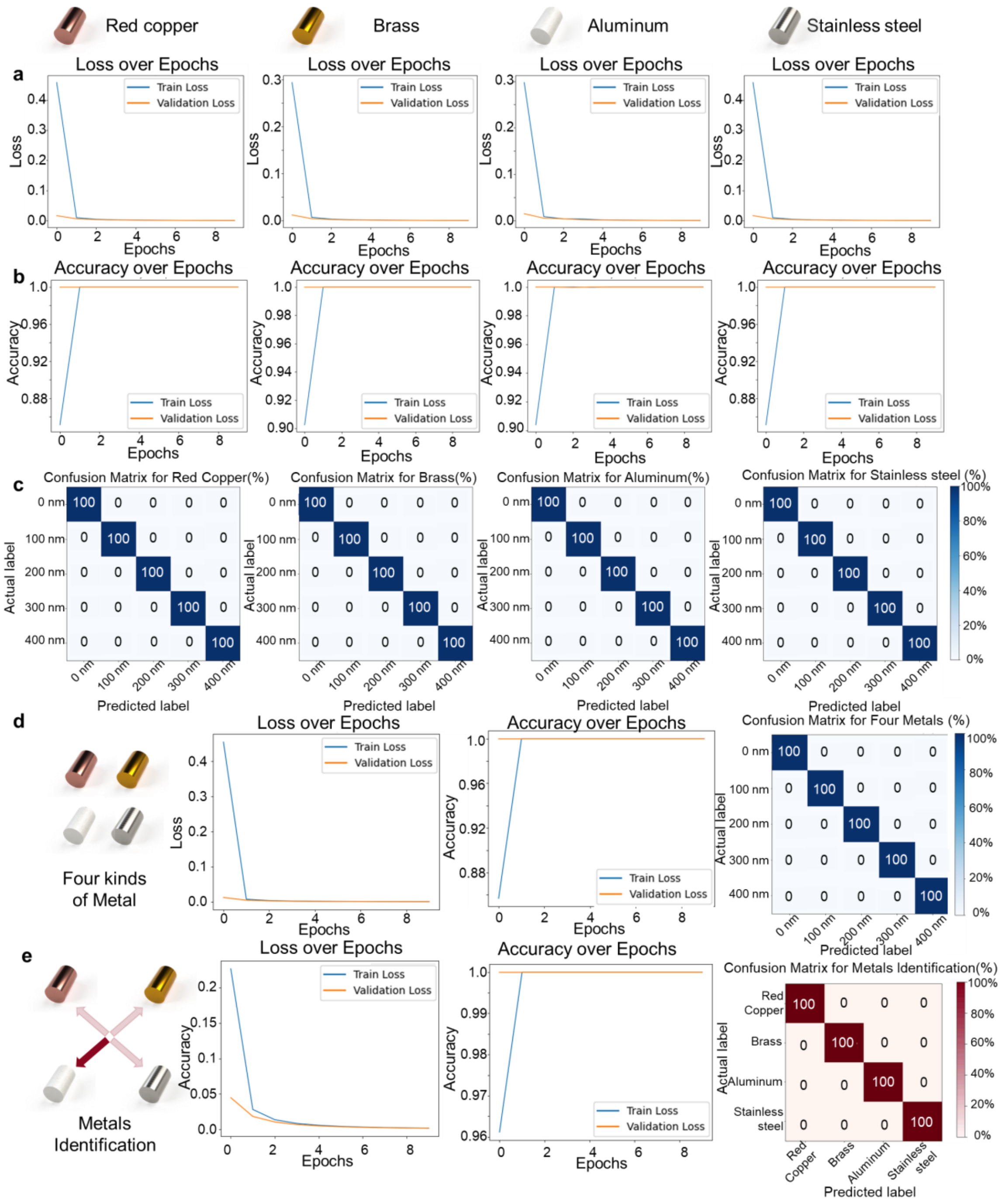

Fig. S17. Experimental results of displacement detection and material identification of different metal materials. a, The loss function curves, **b,** accuracy curves, and **c,** confusion matrices for red copper, brass, aluminum, and stainless steel at 100 nm resolution. **d,** The loss function curves, accuracy curves, and confusion matrices for joint learning the four different metals. **e,** The training loss function curves, recognition accuracy curves, and confusion matrix for material identification.

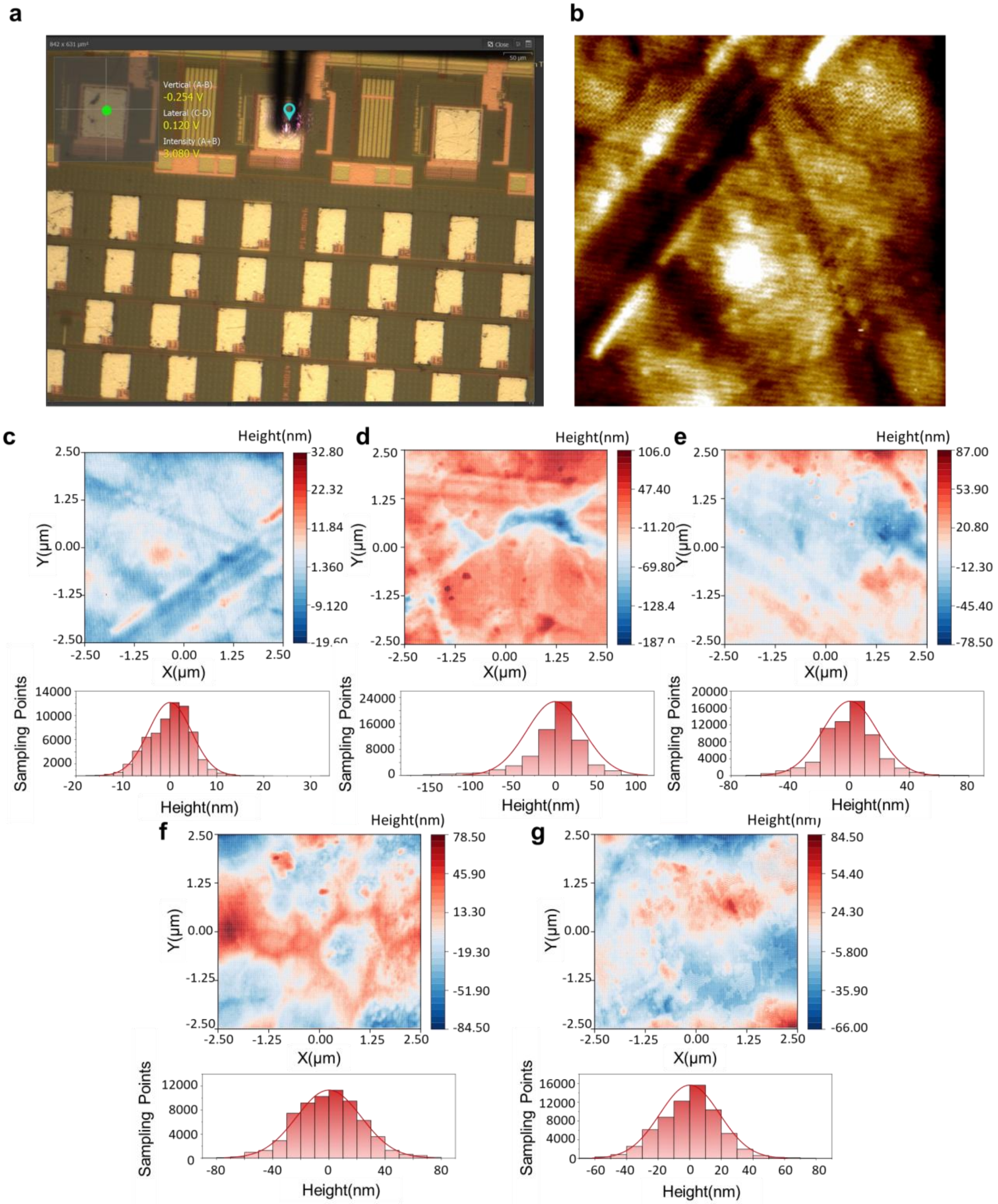

Fig. S18. Surface roughness of the wafer and metal plates measured by atomic force microscopy (AFM). a, Selected sampling area on the wafer surface. **b,** Height distribution within the measured 5 μm × 5 μm region. Two-dimensional surface roughness distributions and height histograms of **c,** Wafer surface. **d,** Stainless steel plate. **e,** Brass plate. **f,** Aluminum plate,and **g,** Red copper plate.

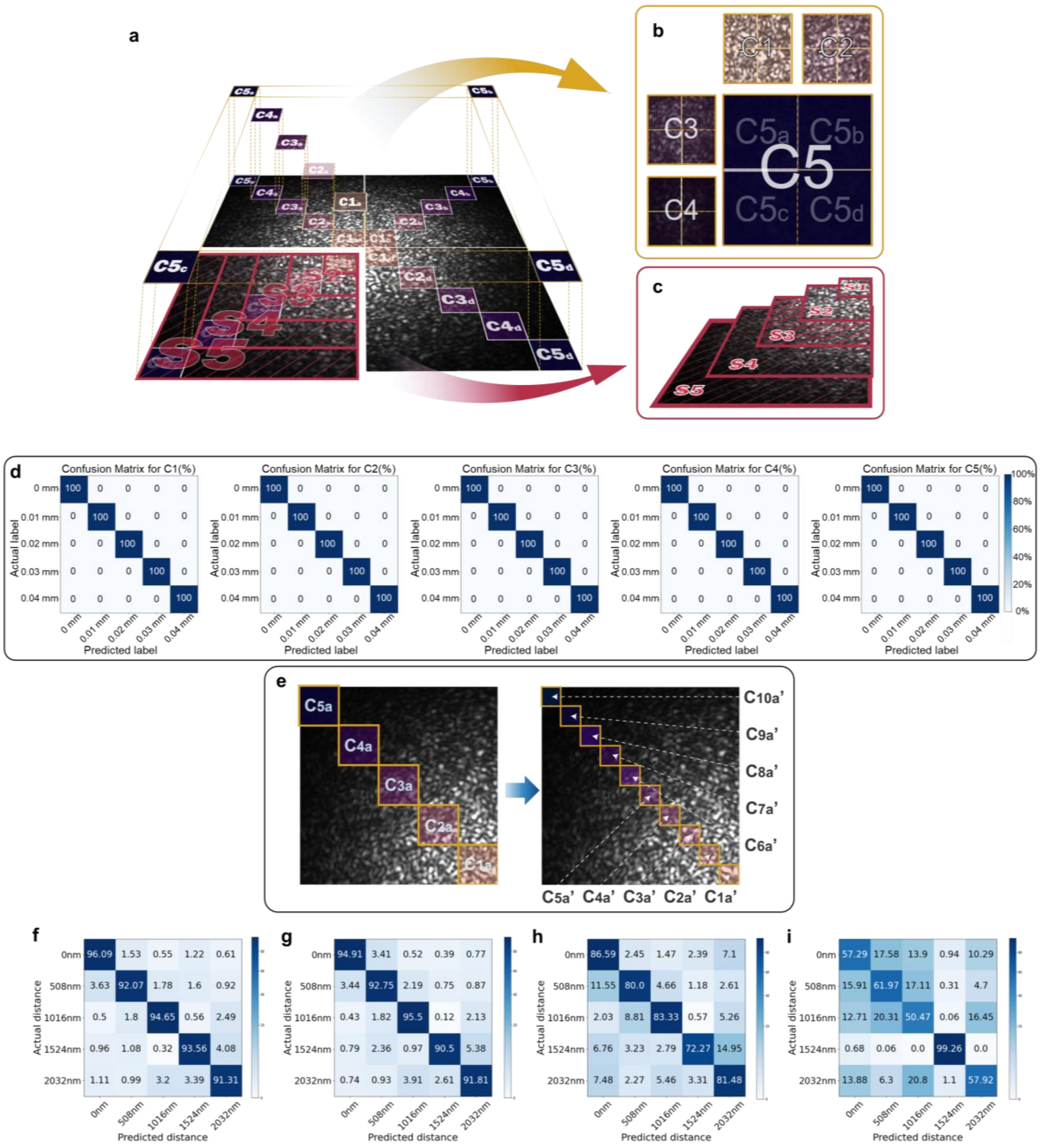

Fig. S19. Compressed sampling speckles for displacement detection. a-c, Different compressed sampling and combination approaches. **d,** The results of displacement detection with a resolution of 0.01 mm using the compressed sampled diffuse speckle field of C1-C5 regions. **e,** Further reduce the sampling area according to the C-type sampling method. Split the upper left quarter of the speckle field into 10×10 small squares and take one of them on the diagonal for training. **f, g,** Confusion matrices for distance detection of the sampled C1a' and C10a' regions, each compressed to a 4×4-pixel size, respectively. **h, i,** Confusion matrices for distance detection of the sampled C1a' compressed to 3×3-pixel and 2×2-pixel size, respectively.

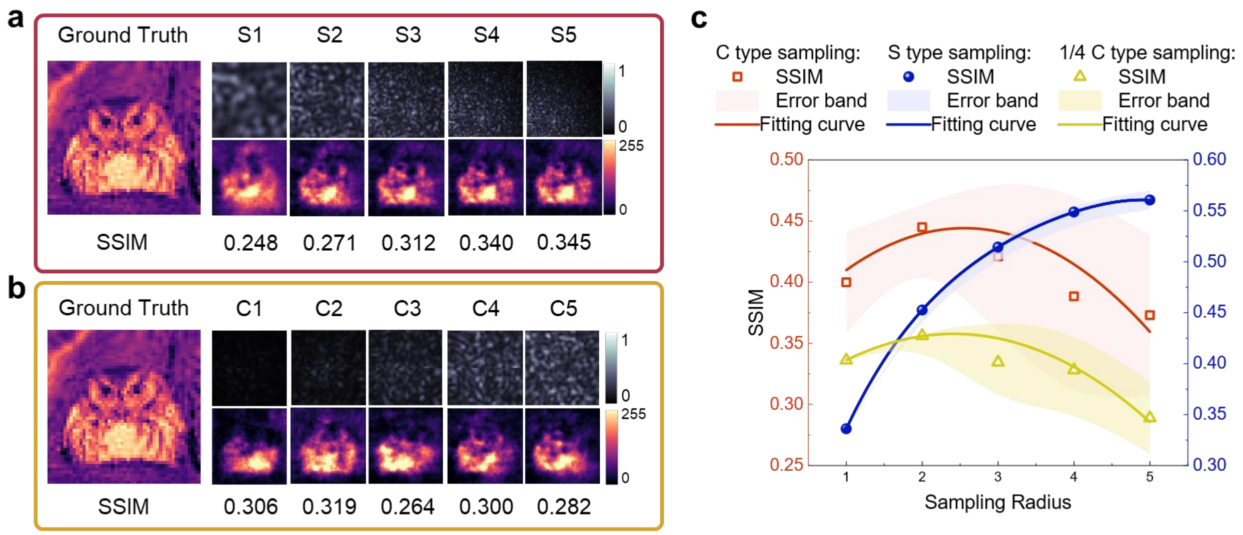

Fig. S20. Compressed sampling speckles for image[11] reconstruction. a, Image reconstruction results for S1-S5 compressive sampled speckles. **b,** Image reconstruction results for C1-C5 compressive sampled speckles. **c,** Reconstructed structural sismilarity curves through C-type sampling, S-type sampling, and 1/4C-type sampling of speckles. The scatter is the actual results, the curves are the fitting results, and the shaded areas are the error bands obtained after several repetitions of the experiment. Red and yellow results correspond to the left axis, blue results correspond to the right axis. The accuracy curves for different compressed sampling regions indicate that different information capacities are included.

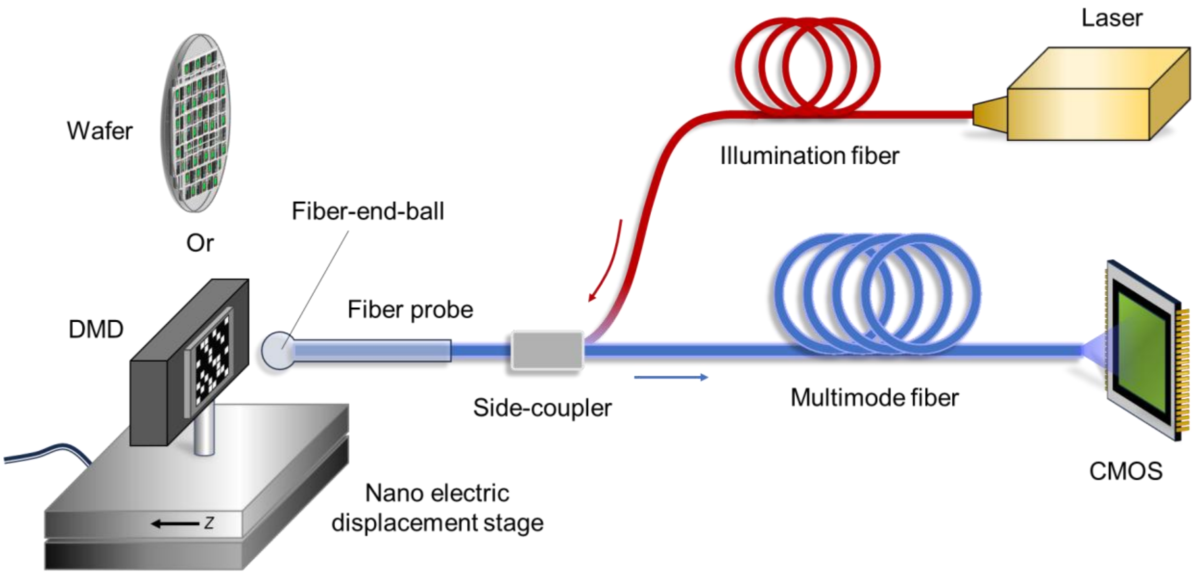

Fig. S21. Structure of the experimental system for nano-displacement detection with multimode fiber. The illumination light from the laser(Connet, CoSF-D-YB-M-1064-PM-FA) is transmitted along the illumination fiber(YOFC, 135/155 0.22/0.46NA). It is injected into the cladding of the multimode fiber through a side-coupler(Lasfiberio, MPSC-2-F-M15/LD330-LD330-P257-1m). In order to increase the detection area, the front end of the multimode fiber is fused to a section of large size triple clad fiber(Nufern, BD-S200/230/660-STN). The probe end of the fiber optic probe is fused with a fiber-end-ball, which homogenizes and collimates the illumination field. The illumination is directed onto a wafer(SMIC), DMD(Texas Instruments, DLP LightCrafter 4500), or other detected target. The detected target is loaded on a nano electric displacement stage(COREMORROW, P18.X300S & New focus, Model 8751-C). The reflected light carries displacement information that is recollected by the fiber-end-ball. The signal light travels backwards along the multimode fiber(Nufern, LMA-GDF-30/250-M) and is ultimately received by the CMOS photoreceptor(Sony, 1/1.8" IMX252 Global shutter CMOS). To further increase the integration of the system, there is no lens or objective between the fiber output end face and the CMOS sensor, only an air layer. The entire optical path is highly integrated without any discrete optical components. The system is fully integrated with special fiber optic devices to achieve all-fiber structure.

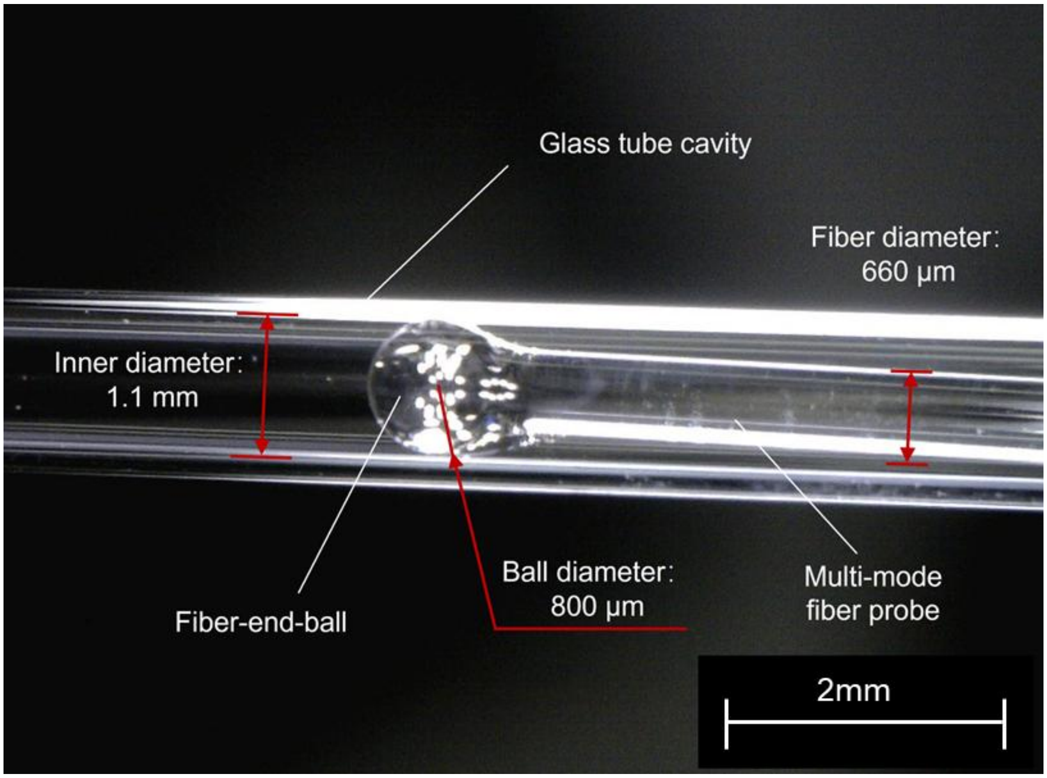

Fig. S22. Micrograph of the multimode fiber probe inserted into a capillary glass tube.

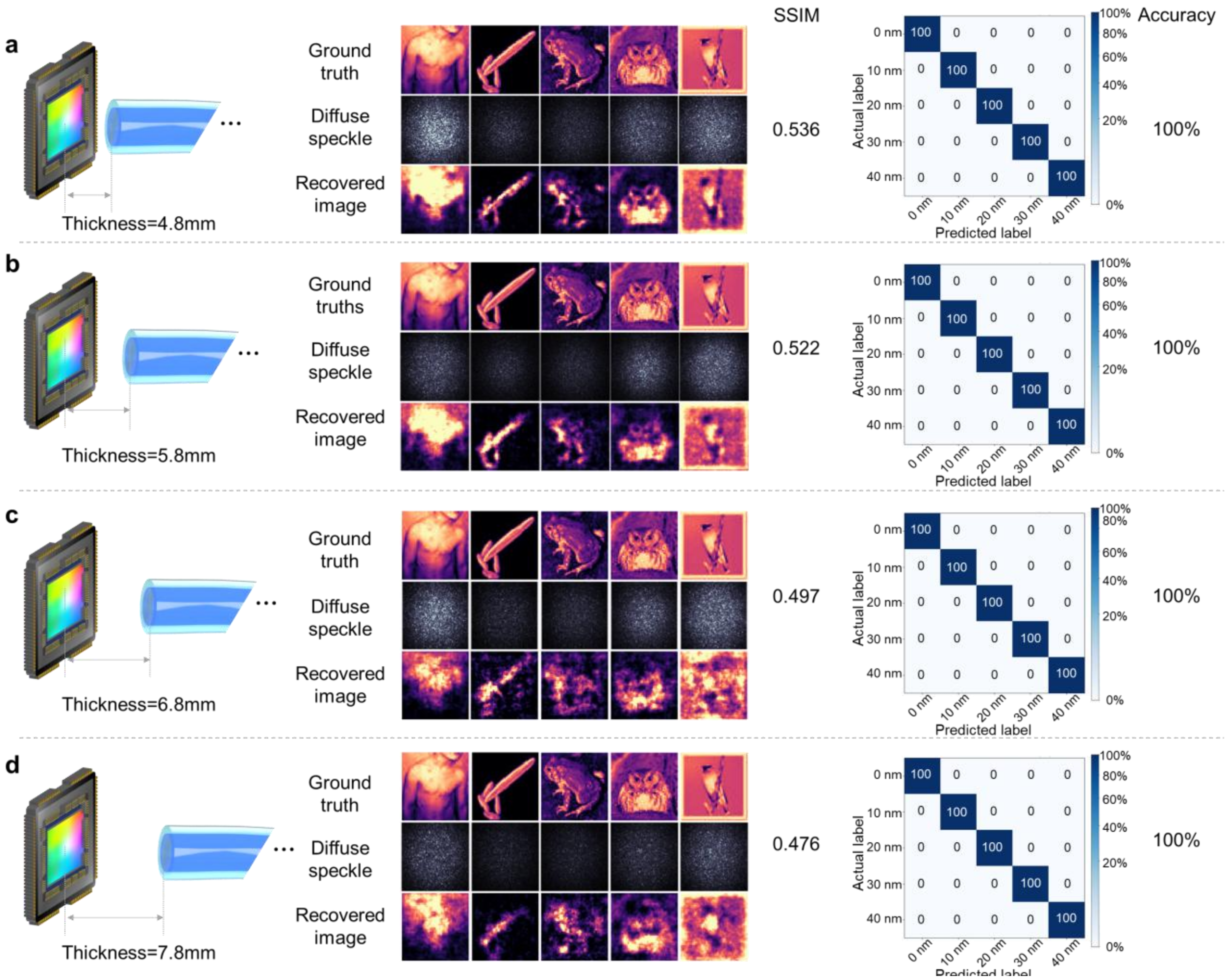

Fig. S23. Diffuse speckles, image reconstruction and displacement recognition results of different air layer thicknesses. Schematic diagram of the output end of the MMF, the acquired diffuse speckle field, the reconstruction results and displacement recognition confusion matrices of images with thicknesses of **(a)** 4.8mm, **(b)** 5.8mm, **(c)** 6.8mm, and **(d)** 7.8mm. The target images are grayscale natural scenes[11-15].

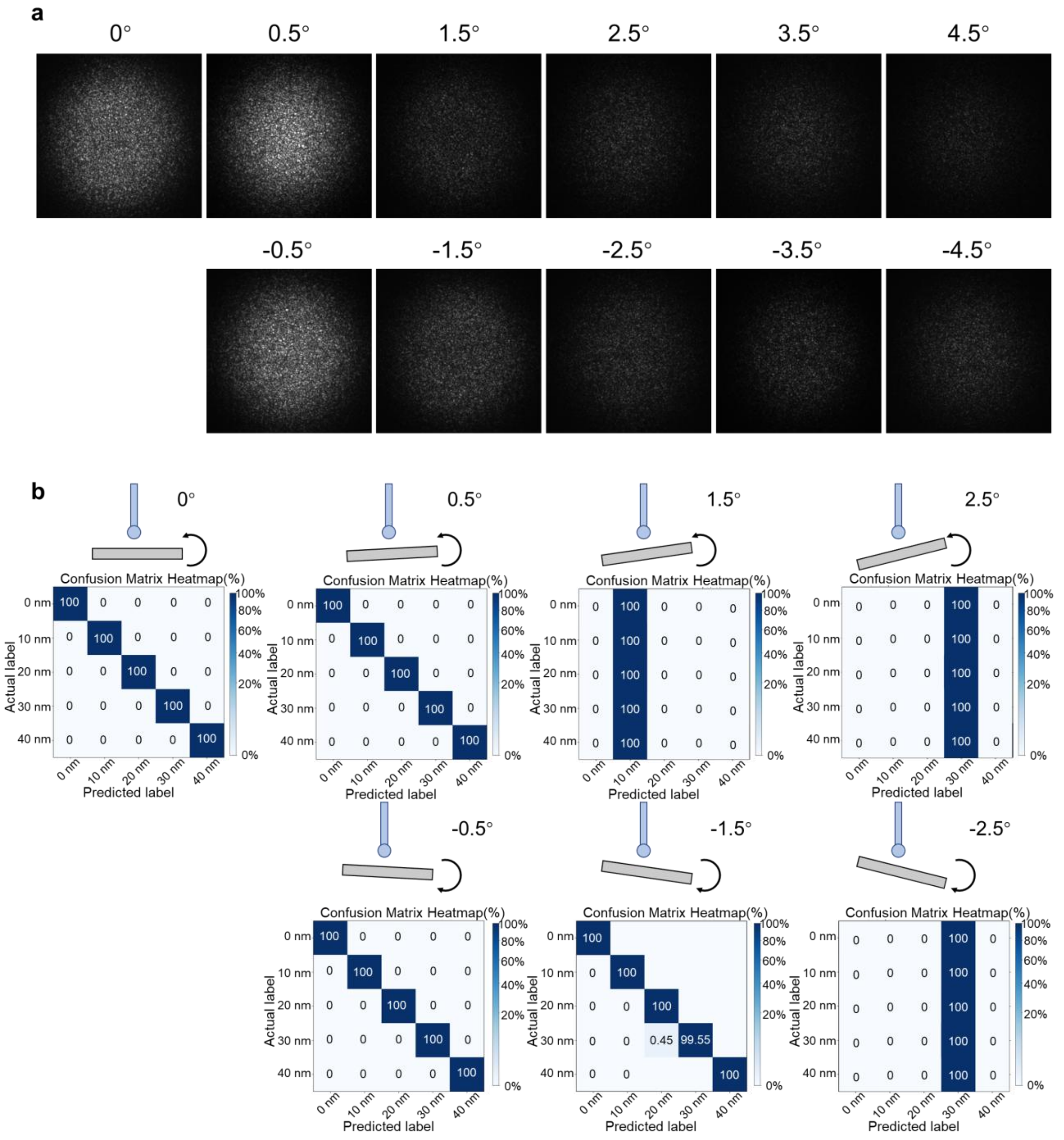

Fig. S24. Diffuse speckle fields captured at the output and confusion matrices for displacement recognition at different tilt angles. a, Diffuse speckle fields captured with the sample normal and fiber axis are misaligned at different angles. **b,** The angle between the wafer sample normal and the fiber axis is 0°, ±0.5°, ±1.5°, and ±2.5°, respectively, showing the results obtained from training the displacement recognition network at each tilt angle.

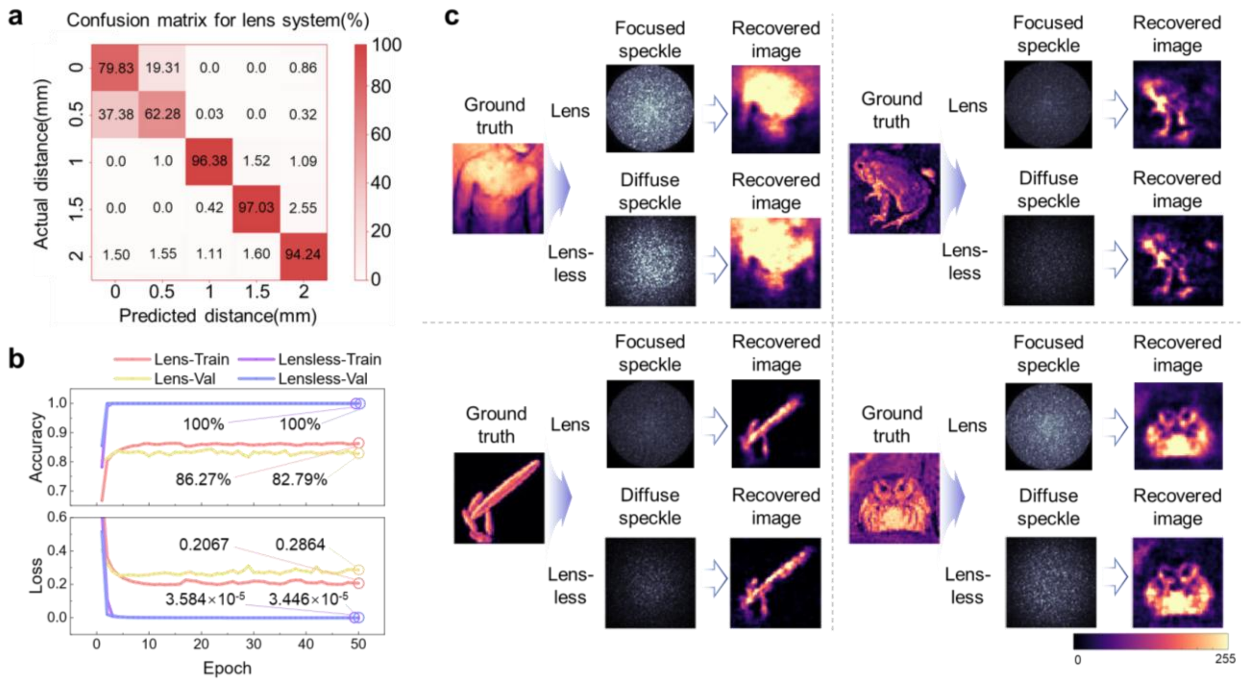

Fig. S25. Comparison of the proposed lensless system with the conventional lens system. a, Distance detection confusion matrix of the lens system. **b,** Accuracy curves and loss curves of the train set and the validation set. **c,** The image reconstruction results of the proposed system and the conventional lens system. The target images are grayscale natural scenes[11-13, 15].

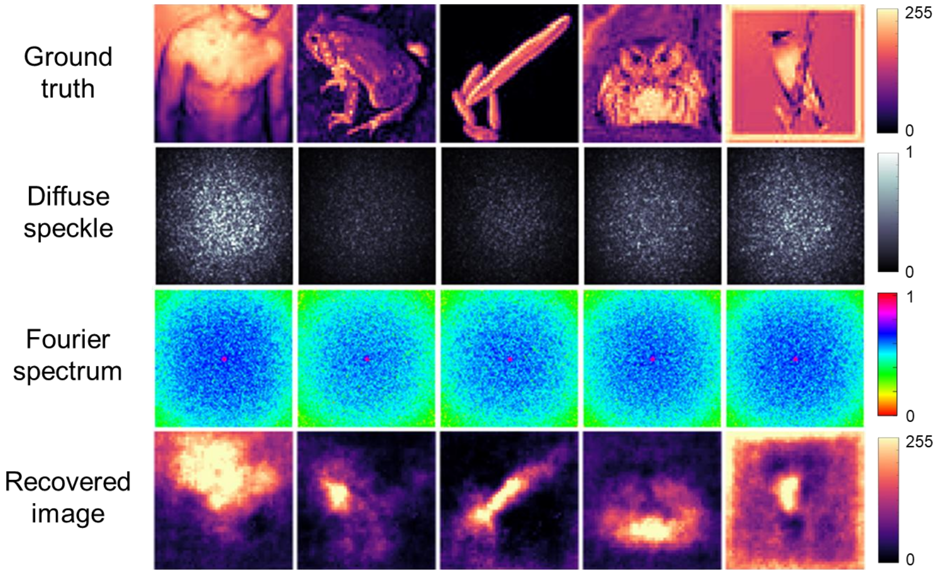

Fig. S26. The frequency-domain amplitude spectrum corresponding to the diffuse speckle field and the reconstruction results from the Fourier spectrum. The target images are grayscale natural scenes[11-15].

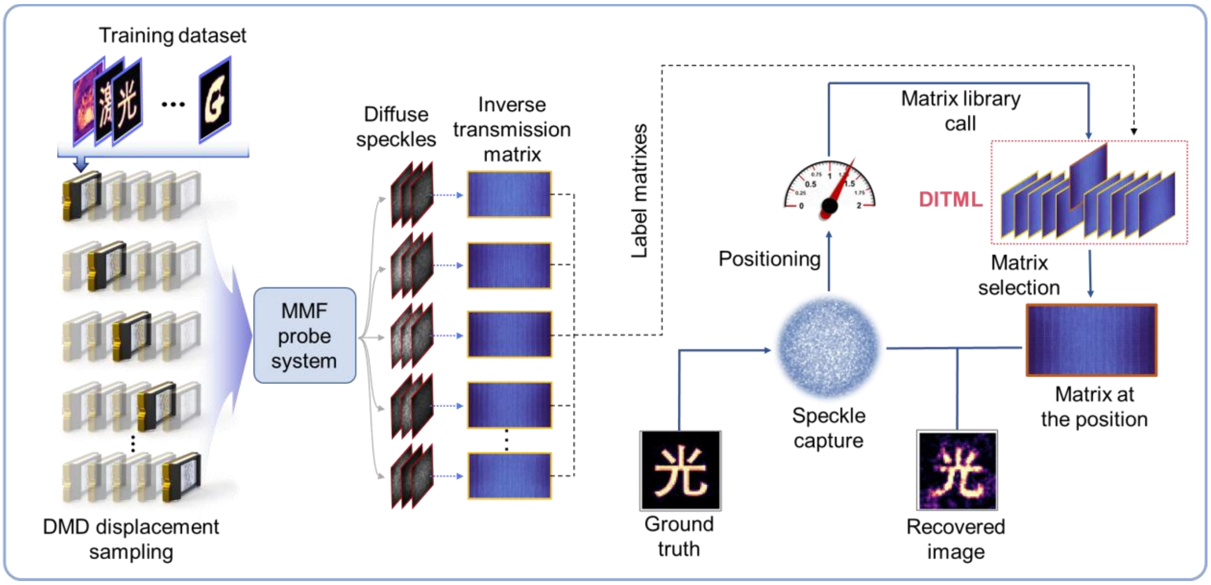

Fig. S27. The overall process of displacement detection and image[11] reconstruction by integrating the VGG displacement recognition network and inverse transmission matrix optimization network.

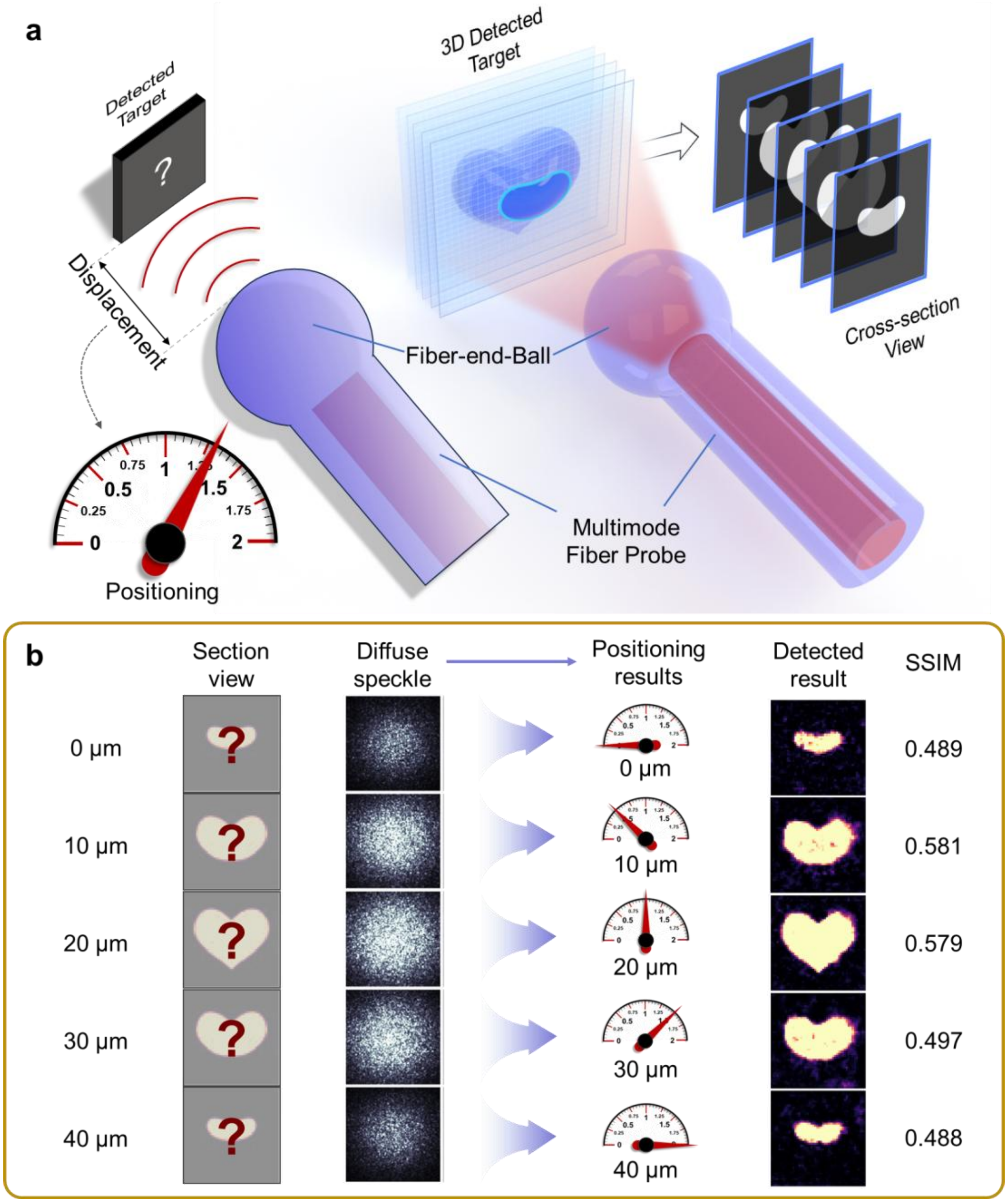

Fig. S28. Positioning and 3D micro structural cross-section reconstruction results. a, Schematic concept of positioning and 3D micro structural cross-section reconstruction. **b,** Results of cross-section reconstruction of heart-shaped three-dimensional structures.

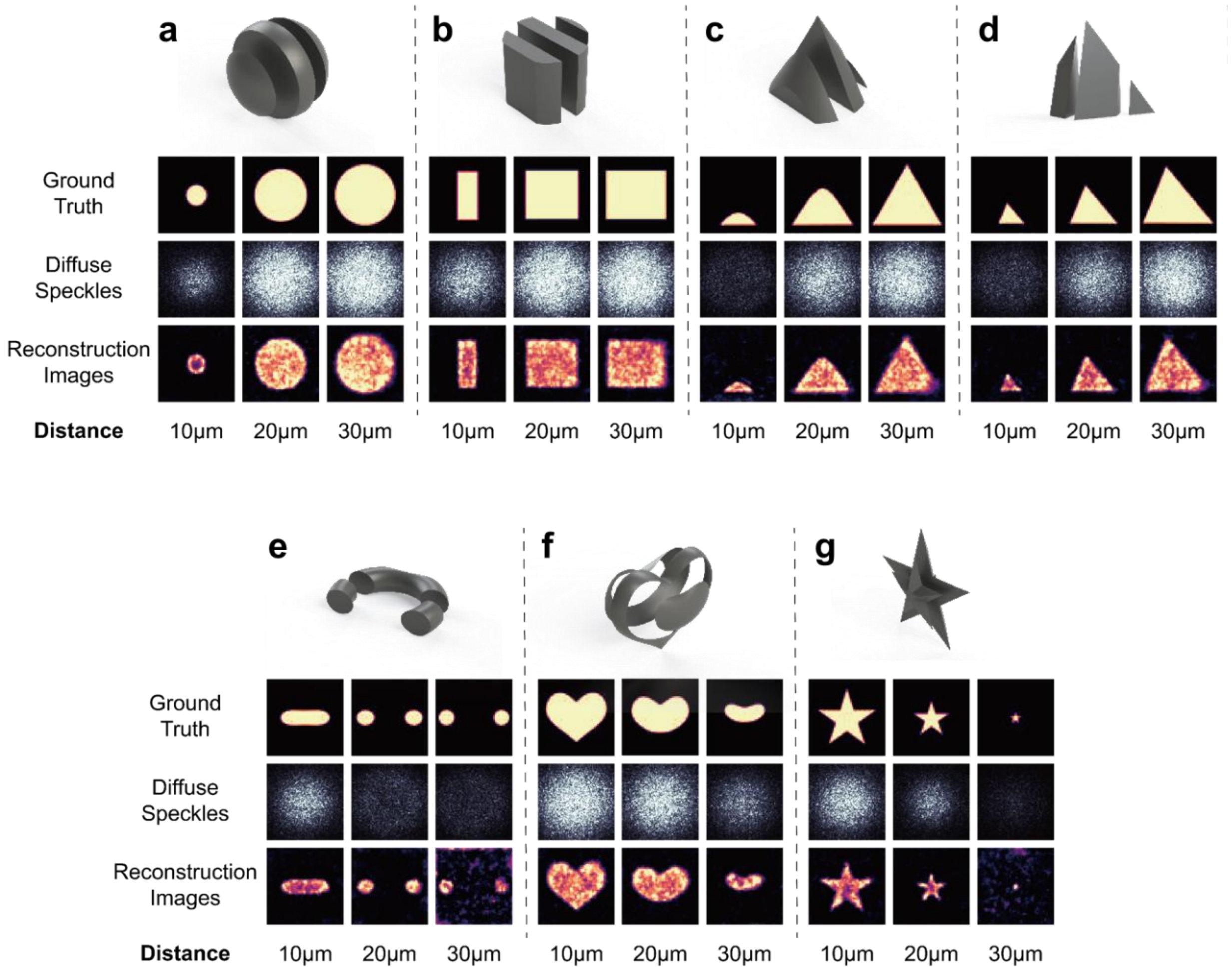

Fig. S29. The results of the detection and reconstruction of simple three-dimensional structures performed by the system. Diffuse speckle fields and reconstructed images resulted from probing cross sections of simple three-dimensional structures such as **(a)** sphere, **(b)** cylinder, **(c)** cone, **(d)** tetrahedron, **(e)** ring, **(f)** heart, and **(g)** star.

Supplementary Table

Table S1. Results of the simulated normalized modes coefficient indicate

Distance/mm	0	1	2	3	4	5	6	7	8	9	10
O_n	11.90	11.53	11.88	11.30	9.43	10.42	9.71	8.94	9.33	9.43	10.04

References

1 Zheng, W. in *2015 Optoelectronics Global Conference (OGC)* 1-7 (IEEE, 2015).

2 Yuan, G. H. & Zheludev, N. I. Detecting nanometric displacements with optical ruler metrology. *Science* **364**, 771-775 (2019).

3 Zuo, C. *et al.* Transport of intensity equation: a tutorial. *Opt. Lasers Eng.* **135**, 106187 (2020).

4 Hopkins, H. The frequency response of a defocused optical system. *Proc. R. Soc. London, Ser. A* **231**, 91-103 (1955).

5 Abrashitova, K. & Amitonova, L. V. Multimode fiber ruler for detecting nanometric displacements. *APL Photonics* **7**, 086103 (2022). https://doi.org:10.1063/5.0089159

6 Hanin, B. & Rolnick, D. How to start training: The effect of initialization and architecture. *Advances in neural information processing systems* **31** (2018).

7 (ed Moran O. Caramazza P., Murray-Smith R. & Faccio D.) (2019).

8 *Google Colaboratory*, <https://colab.research.google.com/#scrollTo=Nma_JWh-W-IF> (2024).

9 Zheng, W. in *2015 Optoelectronics Global Conference (OGC).* 1-7 (IEEE).

10 Goodman, J. W. *Introduction to Fourier optics*. (Roberts and Company publishers, 2005).

11 Wikimedia Commons contributors, File:EasternScreechOwlBillWaller.jpg, Wikimedia Commons,https://commons.wikimedia.org/w/index.php?title=File:EasternScreechOwlBillWaller.jpg&oldid=643429104 (accessed November 19, 2025).

12 Wikipedia contributors, File:Child with chickenpox.jpg, Wikipedia, https://en.wikipedia.org/wiki/File:Child_with_chickenpox.jpg#filelinks (accessed November 19, 2025).

13 Edward Russell, Hog Warts, flickr, https://www.flickr.com/photos/edrussell/122841745/in/photostream/ (accessed November 19, 2025)

14 Saj Sodhi, Cedar Waxwing - Dorsal Shot, flickr, https://www.flickr.com/photos/12466980@N05/2751263737/ (accessed November 19, 2025)

15 Esther Angert, the bacterium Epulopiscium fishelsoni, Cornell University, USA.
