# Supplementary material for: Deep learning and superoscillatory speckles empowered multimode fiber probe for in situ nano-displacement detection and micro-imaging
Source: Nat Commun. 2026 Jan 5;17:1174. doi: 10.1038/s41467-025-67942-8 (PMC12858975; doi:10.1038/s41467-025-67942-8)
Supplement: Supplementary file 2 — Description of Additional Supplementary Files [file 41467_2025_67942_MOESM2_ESM.pdf]

Description of Additional Supplementary Files

Supplementary Movie 1. Multimode fiber output speckle field varies in real time with wafer displacement.
The microscope captures the scene of a wafer detected by the multimode fiber optic probe and the real-time variation of the output speckles captured by a CMOS sensor at displacement resolution of 50 μm, and 10 nm.

Supplementary Movie 2. Flow of Displacement Detection and Image Reconstruction Using DITML.
The process of constructing the Displacement-Inverse Transmission Matrix Library and applying it to the optimization of image transmission quality.
